# Non-canonical role of the SNARE protein Ykt6 in autophagosome-lysosome fusion

**DOI:** 10.1371/journal.pgen.1007359

**Published:** 2018-04-25

**Authors:** Szabolcs Takáts, Gábor Glatz, Győző Szenci, Attila Boda, Gábor V. Horváth, Krisztina Hegedűs, Attila L. Kovács, Gábor Juhász

**Affiliations:** 1 Department of Anatomy, Cell and Developmental Biology, Eötvös Loránd University, Budapest, Hungary; 2 Hungarian Academy of Sciences, Premium Postdoctoral Research Program, Budapest, Hungary; 3 Institute of Genetics, Biological Research Centre of the Hungarian Academy of Sciences, Szeged, Hungary; Stanford University School of Medicine, UNITED STATES

## Abstract

The autophagosomal SNARE Syntaxin17 (Syx17) forms a complex with Snap29 and Vamp7/8 to promote autophagosome-lysosome fusion via multiple interactions with the tethering complex HOPS. Here we demonstrate that, unexpectedly, one more SNARE (Ykt6) is also required for autophagosome clearance in Drosophila. We find that loss of Ykt6 leads to large-scale accumulation of autophagosomes that are unable to fuse with lysosomes to form autolysosomes. Of note, loss of Syx5, the partner of Ykt6 in ER-Golgi trafficking does not prevent autolysosome formation, pointing to a more direct role of Ykt6 in fusion. Indeed, Ykt6 localizes to lysosomes and autolysosomes, and forms a SNARE complex with Syx17 and Snap29. Interestingly, Ykt6 can be outcompeted from this SNARE complex by Vamp7, and we demonstrate that overexpression of Vamp7 rescues the fusion defect of ykt6 loss of function cells. Finally, a point mutant form with an RQ amino acid change in the zero ionic layer of Ykt6 protein that is thought to be important for fusion-competent SNARE complex assembly retains normal autophagic activity and restores full viability in mutant animals, unlike palmitoylation or farnesylation site mutant Ykt6 forms. As Ykt6 and Vamp7 are both required for autophagosome-lysosome fusion and are mutually exclusive subunits in a Syx17-Snap29 complex, these data suggest that Vamp7 is directly involved in membrane fusion and Ykt6 acts as a non-conventional, regulatory SNARE in this process.

## Author summary

SNARE proteins are critical executors of most vesicle fusion events in eukaryotic cells. 4 SNARE domains assemble into a bundle to promote fusion. We have previously shown that Syntaxin 17, Snap29 (contributing 2 SNARE domains) and Vamp7 form the SNARE complex executing autophagosome-lysosome fusion in Drosophila. Surprisingly, one more SNARE protein (Ykt6) is also required in vivo for autophagosome-lysosome fusion. We find that Ykt6 can form a less stable complex with Syntaxin 17 and Snap29 than Vamp7, because Vamp7 outcompetes Ykt6. Ykt6, Vamp7 and Syntaxin 17 all bind to the tethering complex HOPS to promote vesicle fusion. Ykt6 likely plays a non-canonical role in autophagosome-lysosome fusion, because its mutant form (which is thought to be unable to assemble into a fusion-competent SNARE complex) still rescues the fusion defect of ykt6 mutant cells, and it restores viability in mutant animals.

## Introduction

Macroautophagy (hereafter autophagy) is a conserved lysosomal self-degrading and self-renewal pathway in eukaryotic cells. The fusion of autophagosomes with late endosomes/lysosomes is a critical step of autophagy. Fusion of eukaryotic membranes requires several functionally conserved factors including Rab family GTPases, tethering complexes and the appropriate complex of soluble N-methylmaleimide sensitive factor attachment protein receptor (SNARE) proteins. Rab GTPases define membrane identity and recruit the appropriate tethering factors. Tethers bridge the two membranes, and in several cases help the assembly of the SNARE complex, too [1,2]. Most SNAREs are membrane anchored proteins, and all of them contain either one or two alpha helical SNARE motifs. This special coiled coil motif allows these proteins to form heterotetrameric SNARE complexes. Based on the biochemical properties of this motif, SNAREs are classified as Qa, Qb, Qc or R types. Qa, Qb and Qc SNAREs contain a glutamine while R SNAREs contain an arginine in the central (zero) layer of the assembled SNARE bundle. A fusion-competent quaternary SNARE complex contains one of each in the following orientation: Qabc SNARES are present in the first membrane and the complementary R-SNARE is found in the opposite membrane. The formation of a QabcR quaternary trans-SNARE complex promotes membrane fusion. After the fusion, SNAREs remain zippered in the same membrane (referred to as a cis-SNARE complex) until the Nsf-αSNAP complex untangles the bundle, followed by recycling of the monomeric SNAREs [3].

In metazoans, Rab7 and Rab2 are the most important small GTPases mediating autophagosome-lysosome fusion through their interaction with the HOPS heterohexameric tethering complex [4–7]. HOPS not only acts as a tether, it also interacts with the autophagosomal Qa SNARE Syntaxin 17 (Syx17), and helps the assembly of the Syx17-Snap29-Vamp7/8 (Drosophila/mammalian, respectively) Qa-Qbc-R SNARE complex [8–11].

Vamp7 together with Sec22 and Ykt6 belongs to the longin subfamily among R-SNAREs, as all of them have a ~150 aa longin N-terminal domain (LD) [12], which distinguishes these from the brevin subfamily that have shorter N-termini. Sec22 and Vamp7 act in the Golgi to ER transport pathway [13] and late endosomal-lysosomal fusion events [14,15], respectively. However, the role of Ykt6 is less well characterized. In contrast to Vamp7 and Sec22, Ykt6 has no transmembrane domain: it associates to membranes through its double lipid anchor. The C terminus of mammalian Ykt6 contains a CCAIM motif in which the more C-terminal cysteine is constitutively farnesylated, while the other cysteine is reversibly palmitoylated. This reversible post-translational modification promotes a switch between the open and the closed conformations of the protein. Depalmitoylated Ykt6 forms a closed hairpin structure, in which the longin domain folds back to the SNARE motif. As the farnesyl group is hidden in this conformation, the closed form of Ykt6 is soluble, and it becomes membrane-associated in the open, palmitoylated form [16–22]. This cycling between the cytoplasm and membranes makes Ykt6 extremely mobile, as it can travel between different compartments independent of membrane trafficking routes. Due to this high mobility, Ykt6 has an unusually diverse function in various vesicular pathways. Its role was described in the early secretory pathway [23–25], it may also promote secretion [26,27], and finally, yeast Ykt6 interacts with HOPS and plays an important but incompletely understood role in vacuolar fusion events [28–30]. During autophagy, yeast Ykt6 was suggested to promote phagophore assembly and autophagosome formation, rather than fusion [31].

The regulation of metazoan autophagosome-lysosome and yeast homotypic vacuole fusions show some similarities [32]. In yeast cells, fusion is mediated by HOPS and Ypt7/Rab7 [33]. Although vacuolar SNAREs are not conserved (except for the Qb Vti1), all of these show structural and functional similarities to their metazoan analogs. Vam3 is a syntaxin-like Qa, Vam7 is a SNAP-like Qc [34] and finally Nyv1 is a Vamp7-like longin family R-SNARE with a transmembrane domain [35].

One could speculate that metazoan Ykt6 and Vamp7 may also coexist on the surface of lysosomes, and the role of metazoan Ykt6 is unclear. Here we show that Ykt6 is essential for autophagosomal clearance in Drosophila larval fat cells, and that Ykt6 is not functionally redundant with Vamp7. We demonstrate the competition between Ykt6 and Vamp7 for SNARE complex formation with Syx17 and SNAP29, and show that both lysosomal R SNAREs interact with HOPS through their longin domains. Finally, we demonstrate that the zero layer arginine is not essential for Ykt6 function. These findings suggest that Ykt6 acts as a non-conventional, regulatory SNARE in the course of autophagosome-lysosome fusion.

## Results

### Loss of Ykt6 results in autophagosomal fusion defects in Drosophila

The discovery of the machinery that mediates autophagosome-lysosome fusion in metazoans (the Syx17-Snap29-Vamp7/Vamp8 SNARE and HOPS tethering complexes) suggested that the main players of this process have been identified. We identified the Syx17-Snap29-Vamp7 complex as a result of an RNAi screen targeting Drosophila SNARE-encoding genes in fat cells of starved L3 stage larvae [8]. In addition to these three SNAREs, loss of other genes also influenced autophagy. To evaluate these hits we carried out additional experiments by using independent RNAi lines. We induced autophagy by a 4h starvation in genetically mosaic early L3 larvae, which expressed RNAi transgenes in their GFP positive fat cell clones. Interestingly, cells expressing an RNAi line targeting *ykt6* (ykt6 RNAi/1) resulted in accumulation of small, faint 3xmCherry-Atg8a positive autophagic structures, unlike the bigger, brighter dots seen in surrounding GFP negative control cells, and a similar phenotype was seen when we used an independent RNAi line (ykt6 RNAi/2) **(S1A and S1B Fig)**. This phenotype resembled those of *syx17*, *snap29* or *vamp7* loss of function cells that accumulate autophagosomes due to a block of their fusion with lysosomes [8].

**Fig 1:**
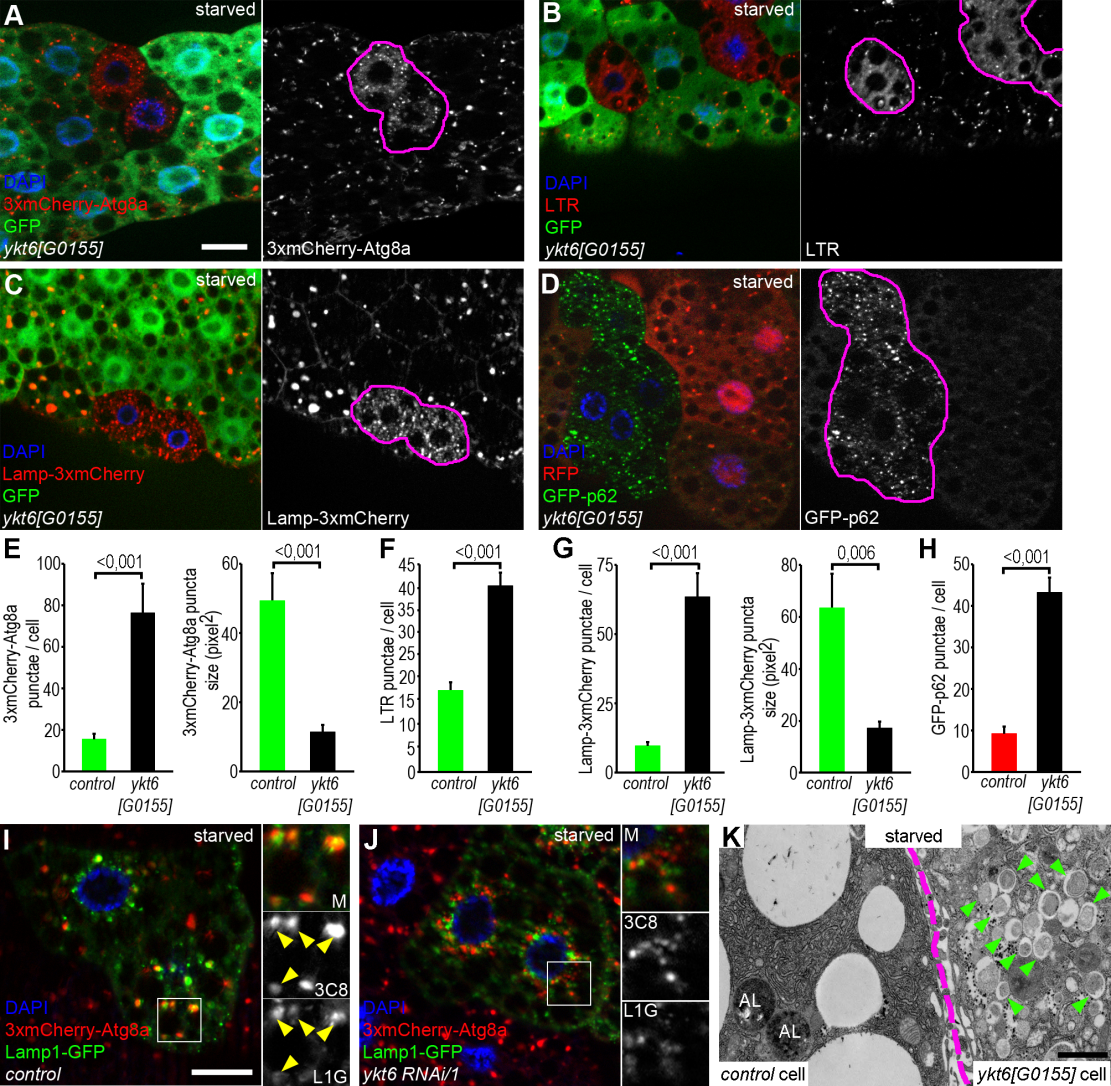
Ykt6 is required for autophagosome-lysosome fusion. (A) Small, faint 3xmCherry-Atg8a positive autophagic structures accumulate in fat cell clones homozygous for *ykt6[G0155]* mutation (GFP-), compared to the surrounding control cells (GFP+). (B) In GFP+ control cells, large LTR positive autolysosomes are visible, while *ykt6* mutant clones (GFP-) contain smaller and more dispersed acidic structures. (C) *ykt6* mutant (GFP-) cells show accumulation of small Lamp-3xmCherry+ lysosomes, compared to controls (GFP+). (D) GFP-p62 levels are low in control cells (RFP+), while this autophagic cargo accumulates in ykt6 mutant clones (RFP-). *ykt6* loss of function cells are encircled in grayscale panels of A-D. (E-H) Quantification of data from A (E), B (F), C (G), and D (H); n = 10. (I, J) Lamp1-GFP lysosomal and 3xmCherry-Atg8a autophagic markers show extensive colocalization (yellow arrowheads) in control cells (I). In contrast, knockdown of ykt6 strongly inhibits the overlap of these two reporters (J). Boxed regions are enlarged in insets, showing merged images (top), red (middle) and green (bottom) channels in I, J. (K) Transmission electron microscopy image from a genetically mosaic larva. Double-membrane autophagosomes (green arrowheads) accumulate and autolysosomes (AL) seen in the control cell are absent from the *ykt6[G0115]* mutant cell. The border between the control and mutant cell is indicated by purple dashed line in K. Scale bars in A and I equal 20 μm for A-D and I, J, respectively. Scale bar in K: 1 μm.

The interesting possibility that Ykt6 might be another R SNARE mediating autophagosome clearance in parallel or independent of Vamp7 prompted us to further investigate the effects of *ykt6* loss. First we wanted to recapitulate the RNAi phenotype by using mutant alleles. We obtained two stocks carrying missense mutations: *ykt6[A]* with a start methionine to isoleucine change, and *ykt6[C]* with a glutamine 62 to arginine change in the longin domain, both of which were isolated in a screen for X-linked lethal mutations that cause morphological and neurophysiological alterations [36]. We also tested a P-element insertion in the protein coding region of *ykt6[G0155]* that truncates the protein after the 76th aa located in the first part of the longin domain (aa 44–131). All homozygous mutants showed lethality in an early developmental stage, so we carried out mosaic analysis. GFP negative mitotic clone cells (homozygous for one of the three *ykt6* loss of function alleles above) accumulated small autophagic structures labelled by 3xmCherry-Atg8a **(Fig 1A and 1E and S1C–S1F Fig)**. Lack of Ykt6 showed a similar phenotype to that seen in *Syx17*, *Snap29*, *Vamp7* or HOPS loss of function cells [8,9], suggesting that Ykt6 is an important regulator of autophagy in Drosophila.

Our next aim was to understand which step of the autophagic process was disturbed in *ykt6* mutant and RNAi cells. We used two different methods to investigate the integrity of the lysosomal/autolysosomal compartment: Lamp-3xmCherry as a lysosomal membrane reporter, and LysoTracker Red, a vital dye that labels acidic (auto)lysosomes. We observed a highly fragmented lysosomal compartment in *ykt6* loss of function cells compared to neighboring controls in both sets of experiments **(Fig 1B, 1C, 1F and 1G)**, again suggesting that cells lacking Ykt6 are defective in autophagosome-lysosome fusion.

We next investigated autophagic degradation using a tubulin promoter-driven GFP-p62 reporter. P62 is a selective autophagosome cargo degraded in autolysosomes. Homozygous *ykt6[G0155]* mutant fat cell clones showed accumulation of GFP-p62 compared to surrounding RFP-negative control cells in both starved **(Fig 1D and 1H)** and well-fed conditions **(S1G and S1H Fig)**, indicating an autophagic degradation (flux) defect.

To confirm that autophagosome-lysosome fusion is perturbed by the absence of Ykt6, we generated Lamp1-GFP (lysosome reporter) expressing control and *ykt6* knockdown fat cell clones, and evaluated its overlap with 3xmCherry-Atg8a positive autophagic structures. As expected, control cells showed large 3xmCherry-Atg8a and Lamp1-GFP double positive autolysosomes (79.5% of 3xmCherry-Atg8a dots, n = 200) **(Fig 1I)**. In contrast, the colocalization was reduced in *ykt6 RNAi* cells (19% and 24% of 3xmCherry-Atg8a dots, n = 200–200, respectively) **(Fig 1J, S2A Fig)**, indicating that these cells are indeed defective in autophagosome-lysosome fusion. Investigating the overlap between 3xmCherry-Atg8a and the lysosomal protease Cathepsin L resulted in similar results: *ykt6[G0155]* mutant cells showed decreased colocalization between these two markers (20.5% of 3xmCherry-Atg8a dots, n = 200) compared to control cells (89% of 3xmCherry-Atg8a dots, n = 200) **(S2B Fig)**. Ultrastructural analysis of *ykt6[G0155]* homozygous mutant cells further confirmed the fusion defect: mutant cells accumulated unfused autophagosomes unlike neighboring control cells **(Fig 1K)**.

Given that Ykt6 is known to function in the secretory pathway, we wanted to exclude the possibility that it affects fusion indirectly via its other role. Syntaxin 5 is a known partner of Ykt6 in yeast and mammalian cells [37] and its loss was shown to impair autophagic degradation [38], so we next analyzed autophagy in Syx5 loss-of-function cells. While the number of 3xmCherry-Atg8a autophagic structures increased in GFP-marked Syx5 knockdown cells, their average size was not significantly different from those in surrounding control cells **(S3A Fig)**. We also generated fat cell clones homozygous for *Syx5[AR113]* mutation, which truncates the 467 aa protein after aa 310 [39]. Interestingly, LTR-positive acidic autolysosomes appeared much brighter in mutant cells than in neighboring control cells **(S3B Fig)**, which likely represent highly acidic autolysosomes that may not be fully functional. Finally, ultrastructural analysis of Syx5 mutant fat cells revealed the presence of autolysosomes containing partially degraded autophagic cargo **(S3C Fig)**. Thus, the different phenotypes of Ykt6 versus Syx5 loss indicate that Ykt6 likely plays a more direct role in autolysosome formation.

### Ykt6 localizes to lysosomes

**Fig 2:**
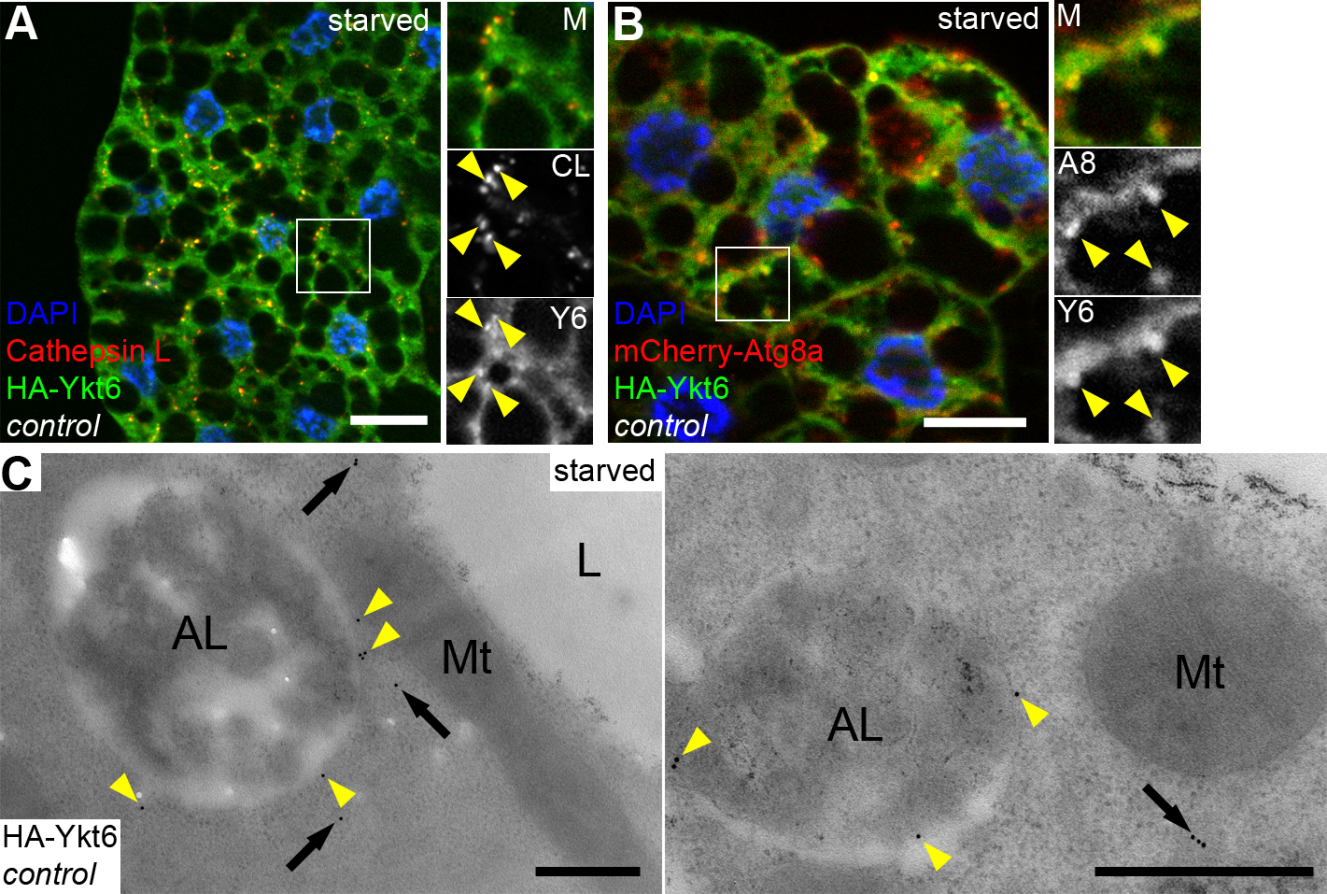
Ykt6 localizes to lysosomes in starved larval fat cells. (A, B) HA-Ykt6 shows obvious colocalization with endogenous Cathepsin-L, a lysosomal hydrolase (A), and also with the 3xmCherry-Atg8a autophagic marker (B). Insets show merged images (top), the corresponding red (middle) and green channels (bottom) enlarged from boxed regions. Yellow arrowheads indicate the overlapping dots. (C) Immunogold labeling shows that Ykt6 is associated with the limiting membrane of autolysosomes (yellow arrowheads) and is also present in the cytosol (arrows). Scale bars: 20 μm (A, B), 0.5 μm (C).

If Ykt6 acts as an R SNARE in the course of autophagosome clearance, it should localize to autophagic or lysosomal membranes. We generated fly lines carrying a UAS promoter driven, N-terminally HA tagged Ykt6 expressing transgenic construct. We crossed these lines with heat inducible Gal4 flies to drive HA-Ykt6 expression in larvae by a short heat shock, followed by immunostainings. HA-Ykt6 showed relatively high diffuse cytoplasmic signal, suggesting that the majority of intracellular Ykt6 is in a soluble form. Additionally, we detected punctate HA-Ykt6 signal that remarkably colocalized with Cathepsin L-positive lysosomes (63.5% of HA-Ykt6 dots, n = 200) **(Fig 2A)**. HA-Ykt6 dots also overlapped with mCherry-Atg8a (48.5% of HA-Ykt6 dots, n = 200) **(Fig 2B)**, a reporter that labels all autophagic structures including autophagosomes and autolysosomes. To explore whether Ykt6 association with lysosomes increases upon autophagy induction, we also looked at well-fed larvae. Indeed, Ykt6 distribution was predominantly diffuse cytosolic under these conditions **(S4A Fig)**.

To confirm that Ykt6 localizes to the limiting membrane of autolysosomes, we performed immuno-EM analysis. Immunogold labeling of HA-Ykt6 revealed that–in addition to its cytosolic pool–Ykt6 preferentially associates with the surface of lysosomes, but not with mitochondria for example **(Fig 2C)**. Our findings that Ykt6 is present on lysosomes raises the possibility that the lysosomal membrane may use two different R SNAREs (Vamp7 and Ykt6) for autophagosome fusion, similarly to yeast vacuolar fusion that also utilizes two R SNAREs: Nyv1 and Ykt6.

We also tested the (auto)lysosomal localization of Ykt6 in Vamp7 mutant fat cells from starved larvae. Starvation is known to increase the number and size of endogenous Cathepsin L-positive (auto)lysosomes during autophagy induction, and loss of Vamp7 leads to fragmentation of the lysosomal compartment, that is, more but smaller Cathepsin L structures are seen in mutants **(S4B and S4C Fig)**. Strikingly, the colocalization of HA-Ykt6 with Cathepsin L-positive lysosomes decreased, with rare partial overlaps (5% of HA-Ykt6 dots, n = 200) **(S4B Fig)**.

Of note, we did not see any change in the expression levels of either Ykt6 or Vamp7 proteins during starvation-induced autophagy and expansion of the lysosomal system, using a previously published anti-Vamp7 antibody and our newly generated anti-Ykt6 antisera, the specificity of which we verified on lysates of wild type and *ykt6* RNAi larvae **(S4D and S4E Fig)**.

### Ykt6 competes with Vamp7 for SNARE complex formation

**Fig 3:**
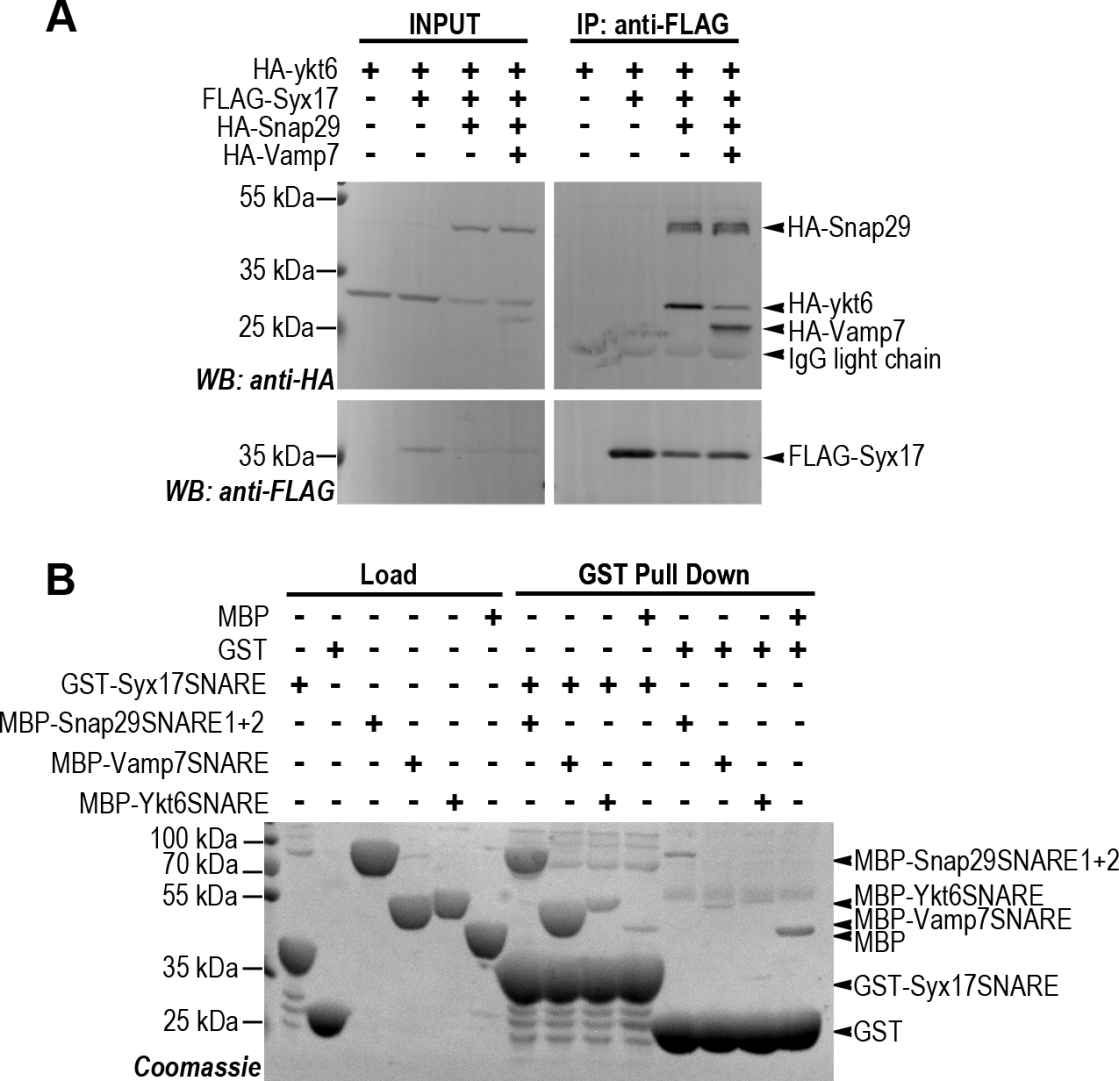
Ykt6 can form a SNARE complex with Syx17 and Snap29. (A) Co-IPs from cultured Drosophila cell lysates expressing tagged SNARE proteins. Ykt6 interacts with the immobilized Syx17 only in the presence of co-expressed Snap29. This interaction is highly reduced in the presence of overexpressed Vamp7. Please note that SNAREs were expressed without their transmembrane domains. (B) GST pull down experiments with recombinant SNARE motifs of Syx17, Vamp7, Ykt6 and Snap29. Both MBP-Snap29^SNARE1+2^ and MBP-Vamp7^SNARE^ strongly bind to immobilized GST-Syx17^SNARE^. MBP-Ykt6^SNARE^ shows weak binding to Syx17. GST serves as negative control.

To further understand the role of Ykt6 during autophagosome-lysosome fusion we performed co-immunoprecipitation (co-IP) and pulldown experiments. First we tested whether Ykt6 could form a classical quaternary SNARE complex with Syx17 and Snap29. No interaction was observed between FLAG-Syx17 and HA-Ykt6 in co-IP experiments from lysates of cultured Dmel2 cells transiently expressing these proteins **(Fig 3A)**. In contrast, HA-Ykt6 showed a strong interaction with FLAG-Syx17 when co-expressed with HA-Snap29, suggesting that Ykt6 can participate in a SNARE complex as an R SNARE. However, the loss of either Vamp7 or Ykt6 perturbs autophagosome-lysosome fusion, so it was not clear which of these proteins function as a bona fide R SNARE during fusion. We tested this in cells expressing all four SNARE proteins. Interestingly, HA-Vamp7 outcompeted HA-Ykt6 from the FLAG-Syx17 containing SNARE complex **(Fig 3A)**, suggesting that the Syx17-Snap29-Ykt6 complex is less stable than the Syx17-Snap29-Vamp7 complex. This is in line with previous yeast experiments showing that Ykt6 can form a complex with vacuolar Q SNAREs in vitro, but the formation of this complex is prevented by Nyv1 [40]. To further explore the interaction between Ykt6 and subunits of the autophagosomal SNARE complex, we carried out pull down experiments with recombinantly expressed GST tagged, full length Ykt6. We observed that Ykt6 binds to endogenous Syx17 but not to overexpressed GFP-Vamp7 from L3 larval lysates **(S5A and S5B Fig)**.

We next purified recombinant MBP- or GST-tagged SNARE motifs of Syx17, Vamp7, Ykt6 and MBP tagged full length Snap29, and carried out pulldown experiments **(Fig 3B)**. Both MBP-Snap29 and MBP-Vamp7 SNARE motifs bound strongly to the GST-tagged Syx17 SNARE motif. We also detected a weaker binding between GST-Syx17 and MBP-Ykt6 SNARE motifs. This also suggests that the Syx17-Snap29-Ykt6 SNARE complex is less stable than the Syx17-Snap29-Vamp7 bundle, explaining how Vamp7 can outcompete Ykt6.

For better understanding why Ykt6 forms an unstable SNARE complex, we generated a homology model by predicting the structures of Syx17-Snap29-Vamp7/Ykt6 complexes in silico. We used the crystal structure of the human Syntaxin17-Snap29-Vamp8 [41] as a template for aligning the SNARE motifs of these Drosophila proteins **(S3C Fig)**. The structure of Vamp7 fits very well into the Syx17-Snap29 complex, and these proteins likely form a tight quaternary SNARE bundle **(see examples in S3D and S3F Fig)**, in line with our previous functional and co-IP analyses [8]. In contrast, the aligned Ykt6 SNARE motif shows charge changes and clashes with the Syx17 SNARE motif at positions Y186 and N193 **(S3E and S3G Fig)**. These predicted models also support our biochemical data that the Syx17-Snap29-Ykt6 complex is less stable than Syx17-Snap29-Vamp7.

### Vamp7 acts downstream of Ykt6 during autophagosome-lysosome fusion

**Fig 4:**
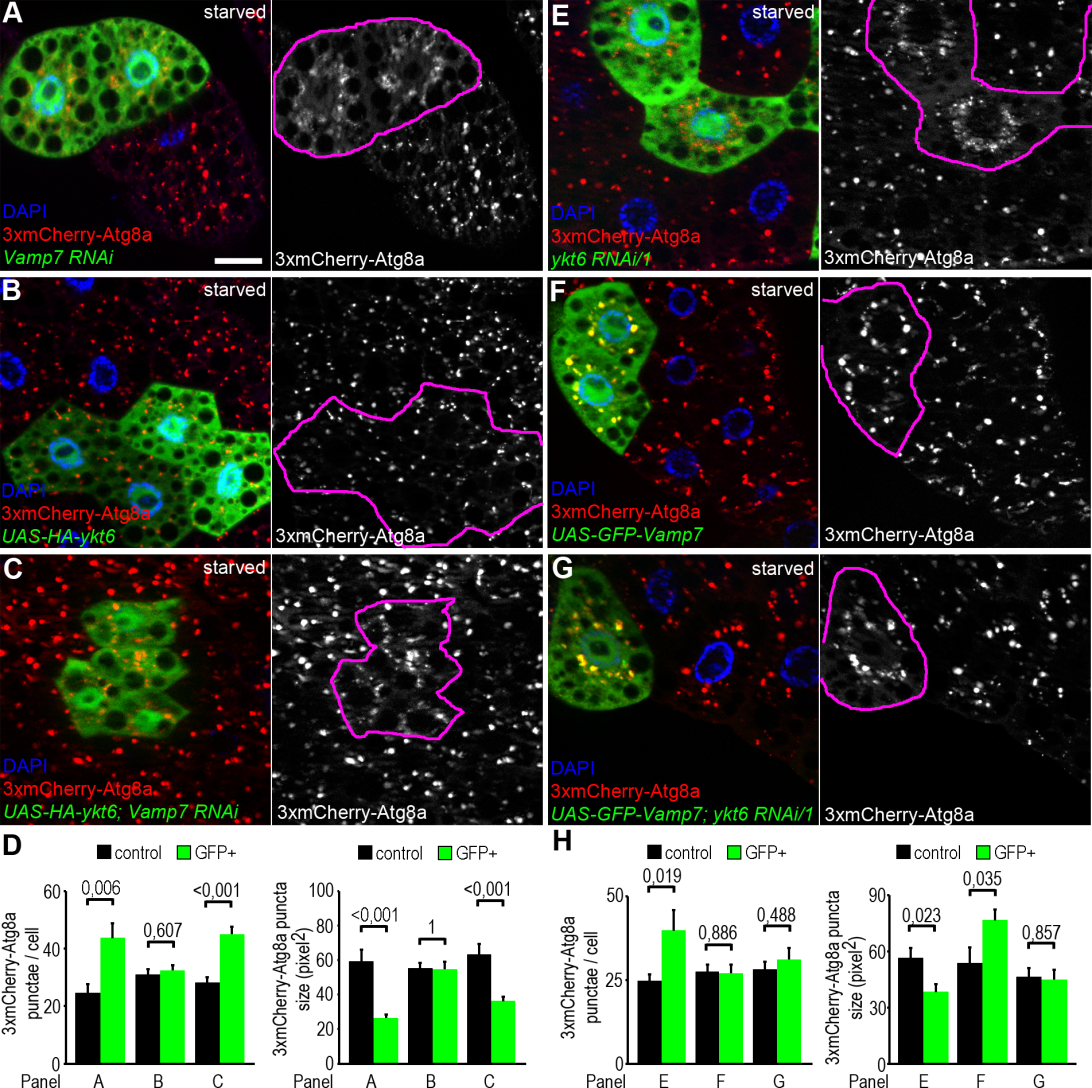
Genetic interactions between Ykt6 and Vamp7. (A-H) Analysis of 3xmCherry-Atg8a in fat cell clones of starved larvae. Knockdown of Vamp7 leads to the accumulation of small faint 3xmCherry-Atg8a positive autophagosomal clusters near the nucleus of GFP+ RNAi cells, as opposed to the big bright autolysosomes seen in controls cells lacking GFP (A). Please note that genetically manipulated cells are encircled in grayscale panels in this and all subsequent images. HA-Ykt6 expression in GFP-labeled clones does not influence the punctate 3xmCherry-Atg8a signal (B), and co-overexpression of Ykt6 in *Vamp7* RNAi cells does not suppress the Vamp7 loss-induced accumulation of autophagosomes (C). Statistical analysis of data from panels A-C (D); n = 10. Knockdown of *ykt6* in GFP-labeled cells also produces perinuclear accumulation of 3xmCherry-Atg8a positive autophagosomes, similar to *Vamp7* RNAi (E, compare to A). Expression of GFP-Vamp7 slightly increases the size of mCherry-Atg8a positive autolysosomes (F). Co-overexpression of Vamp7 rescues the size of 3xmCherry-Atg8a positive autophagic structures in *ykt6* RNAi cells (G). Note that GFP-Vamp7 clearly colocalizes with 3xmCherry-Atg8a in both control and *ykt6* RNAi cells in panels F and G. (H) Statistical analysis of data from panels E-G; n = 10. Scale bar: 20 μm (A-C, E-G).

The co-occurrence of Vamp7 and Ykt6 on lysosomal membranes raises the possibility that these two R SNAREs act redundantly. Although this is a tempting speculation, our findings that Vamp7 can outcompete Ykt6 from the autophagosomal SNARE complex, and that single Vamp7 or Ykt6 mutations cause severe autophagic defects make this hypothesis unlikely. We thus reasoned that Ykt6 and Vamp7 have a separate function during autophagosome-lysosome fusion. We performed epistasis analysis to test the hierarchy between Vamp7 and Ykt6 during the fusion process. RNAi silencing of either Vamp7 or Ykt6 resulted in a highly similar phenotype: mostly perinuclear accumulation of small, faint 3xmCherry-Atg8a positive autophagosomes **(Fig 4A, 4D, 4E and 4H; S1A and S1B Fig)**, as expected. Next we generated fat cell clones overexpressing HA-Ykt6 alone or together with the Vamp7 RNAi transgene. Elevated level of Ykt6 did not change the number and size of mCherry-Atg8a punctae in either case **(Fig 4B–4D)**. In line with this, transient expression of HA-Ykt6 failed to restore the number of LysoTracker positive autolysosomes in fat cells of Vamp7 mutant larvae **(S6A–S6D Fig)**.

We also generated fat cell clones overexpressing GFP-Vamp7 alone or together with ykt6 RNAi transgenes. Interestingly, overproduction of Vamp7 increased the size of autophagic 3xmCherry-Atga structures **(Fig 4F and 4H)**. Importantly, overexpression of Vamp7 restored the number and size of 3xmCherry-Atga structures in Ykt6 RNAi cells **(Fig 4G and 4H; S6E and S6F Fig)**.

To further analyze the epistatic effect of Vamp7 over Ykt6, we generated fat cell clones homozygous mutant for *ykt6[G0155]* in larvae that overexpress GFP-Vamp7 in all fat cells. In accordance with the previous RNAi data, Vamp7 overexpression increased the size of mCherry-Atg8a positive autophagic structures in ykt6 mutant cells, while these cells still remained smaller than the surrounding control ones **(S6G Fig)**. We also tried to rescue the early lethality of *ykt6[G0155]* mutants by ubiquitously overexpressing GFP-Vamp7, but without success.

These results suggest that Vamp7 acts downstream of Ykt6 during autophagosome-lysosome fusion, and increased expression of Vamp7 can compensate for the autophagic but not for other defects of Ykt6 loss of function cells and animals.

### Ykt6 interacts with HOPS through multiple binding sites

What could be the role of a less stable Syx17-Snap29-Ykt6 SNARE complex? Although we cannot completely rule out that Syx17-Snap29-Ykt6 might form a fusion competent SNARE complex, this is unlikely because Vamp7 mutants show a severe autophagosome fusion defect [8], which is not suppressed by Ykt6 overexpression. Ykt6 is essential for vacuole fusion in yeast, but previous studies already raised the possibility that its role is independent of being part of a classical, fusion-competent SNARE complex. Dietrich and colleagues suggested that Ykt6 functions as a palmitoyl transporter and facilitates the palmitoylation of the fusion regulator Vac8, and blocking Vac8 palmitoylation inhibits vacuole fusion [18,42]. They also demonstrated that this palmitoylation occurs at an early time point during vacuole fusion and Ykt6 leaves the fusion site before the completion of fusion. Additionally, McNew and colleagues demonstrated that Ykt6 can mediate membrane fusion only if it is attached to a C terminal transmembrane domain, but not via its natural lipid anchors [43]. These data altogether suggest that Ykt6 has a regulatory role in yeast vacuole fusion.

During the membrane fusion process, SNAREs not only pull the two membranes close to each other to achieve fusion, but they also interact with multisubunit tethering complexes [2]. Others and we showed earlier that Syx17 binds to the HOPS tethering complex during autophagosome-lysosome fusion [9,11]. The SNARE-tether interaction is not necessarily restricted to the SNARE domain: several SNAREs can bind to the appropriate tether through their long N-terminal domains. For example, the vacuolar syntaxin Vam3 can bind to HOPS through its N terminal Habc domain in yeast [44]. Both Ykt6 and Vamp7 have an N-terminal longin domain, but the role of this motif in fusion is still unexplored. To evaluate whether the N-terminal domain (NTD, including the Habc) of Syx17 or the longin domains (LDs) of Ykt6 and Vamp7 can bind to HOPS, we purified the recombinant GST-tagged LDs of Ykt6 and Vamp7, the NTD of Syx17, and full-length Ykt6. In GST pulldown experiments using larval lysates, the LDs of both Ykt6 and Vamp7 as well as the NTD of Syx17 bound to the HOPS subunit myc-Dor/Vps18 **(S7A and S7B Fig)**. Additionally, even endogenous Carnation/Car (Vps33A ortholog) could be clearly detected in pulldowns of full-length Ykt6-GST **(S7A Fig)**. These data raise the possibility that Ykt6 has multiple HOPS interaction sites: its LD probably binds to the core subunit Dor/Vps18, while its SNARE domain likely binds to Car/Vps33A. These results fit well with previous data showing that HOPS subunits interact with various regions of SNARE proteins, but the SNARE chaperoning subunit Vps33 binds only the SNARE bundle [44–46].

To our knowledge, this is the first report of a physical interaction supporting a role for the LDs of R SNAREs in the regulation of vesicle fusion. Binding of the LDs of both Ykt6 and Vamp7 to a core HOPS subunit might be a novel regulatory step of HOPS-mediated vesicle tethering. This also raises the possibility that all LD SNAREs might interact with a multisubunit tethering complex through their LDs. In fact, yeast Sec22 also binds to the ER-Golgi tether Dsl1 complex [47], although the involvement of its LD in this interaction was not tested.

### The zero layer arginine is dispensable for Ykt6 function

Since Ykt6 forms a conventional (albeit perhaps not very stable) SNARE complex with Syx17 and Snap29 and it can be outcompeted by Vamp7, we speculated that Syx17-Snap29-Ykt6 complex may not zipper properly. The zippered SNARE complex contains 16 layers, which stabilize the bundle. Unlike the flanking 15 hydrophobic layers, the central layer (called zero layer) is composed of ionic residues: three glutamines (3Q) and one arginine (1R) as a general rule [48]. In line with the importance of the central ionic residue, an RQ amino acid change in the zero layer of yeast Sec22 leads to cell growth and ER-Golgi trafficking defects [49]. Similarly, changing the zero layer Q to R perturbed Syntaxin 17 function in a mitochondrial protein turnover pathway. Interestingly, this vesicle transport route could be restored by simultaneously mutating Vamp7’s R to Q [50]. In other reports, however, an RQ mutation of the R SNARE did not disrupt the assembly and function of a yeast exocytic complex [51], a double QE mutation of the zero layer in both SNARE domains of a botulinum neurotoxin E resistant SNAP25 was functional in exocytosis assays in multiple human cells [52], and QR mutant syntaxin expression could rescue the viability and locomotor defects of *syntaxin* mutant C. elegans [53]. Nevertheless, as yeast cells carrying an RQ substitution in the zero layer of Ykt6 are lethal just like the null mutant [29], it indicates that the zero layer arginine is critical for Ykt6 function in yeast.

**Fig 5:**
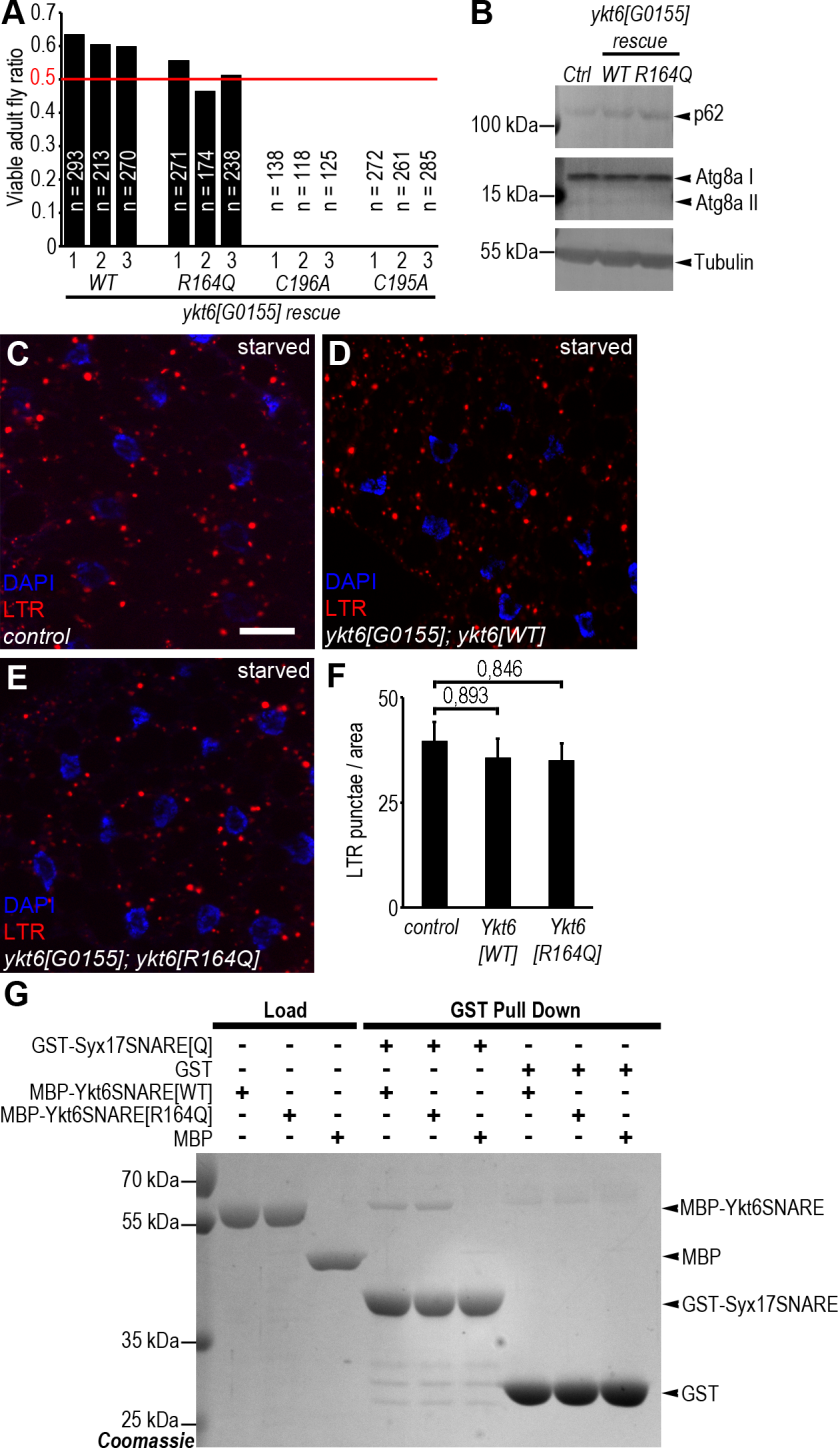
The R164Q amino acid change in the zero layer does not disrupt Ykt6 function. (A) Quantification of in vivo rescue experiments with wild type (WT) and zero layer (Arg 164 to Gln/R164Q), farnesylation (Cys 196 to Ala/C196A) or palmitoylation (Cys 195 to Ala/C195A) site mutant *ykt6* transgenes. *ykt6[R164Q]* fully rescues the early lethal phenotype of *ykt6[G0155]* mutants similar to *ykt6[WT]*. In contrast, *ykt6[C196A]* and *ykt6[C195A]* fail to rescue lethality: no viable larvae or adults were detected from these genotypes. Three transgenes with independent chromosomal insertion sites were evaluated per genotype. Red line marks the expected Mendelian ratio of the genotypes of interest. (B) Similar to wild type flies, *ykt6[G0115]* mutants rescued with WT or R164Q transgenes do not accumulate lipidated Atg8a II and p62. (C-E) Punctate LTR+ autolysosomal staining is similar in fat cells of starved control (C) and *ykt6[G0155]* mutant larvae rescued with expression of WT (D) or R164Q mutant (E) Ykt6 expression. (F) Quantification of data from C-E; n = 10. (G) In pulldown experiments, GST-Syx17^SNARE^ binds with the same affinity to both wild type and R164Q mutant forms of Ykt6 SNARE domain. GST serves as negative control. Scale bar in C is 20 μm for C-E.

We thus carried out genetic rescue experiments using *ykt6* transgenes encoding wild type (WT), zero ionic layer mutant with an R164Q change, and palmitoylation or farnesylation site mutants with C195A or C196A changes, respectively. In human cells, the non-farnesylated mutant is unable to associate with membranes, and the non-palmitoylated mutant is defective in proper subcellular localization [19]. Strikingly, both the *ykt6[WT]* and *ykt6[R164Q]* transgenes fully restored the viability of *ykt6[G0155]* mutant animals, unlike *ykt6[C195A]* and *ykt6[C196A]* **(Fig 5A)**. Moreover, no difference was seen in the levels of p62 and Atg8a proteins between control and *ykt6* mutants rescued with either *ykt6[WT]* or *ykt6[R164Q]* in western blots **(Fig 5B)**. Finally, both *ykt6[WT]* and *ykt6[R164Q]* restored punctate LysoTracker Red autolysosomal staining of starved fat cells on a *ykt6[G0155]* mutant background **(Fig 5C–5F)**. Note that *ykt6[G0155]* mutants and animals rescued with *ykt6[C195A]* or *ykt6[C196A]* could not be evaluated in these tests because of their lethality during early developmental stages.

We also wanted to know if there is any difference between WT and R164Q mutant Ykt6 in their interactions with the other fusion factors Syx17 and HOPS. In a GST pull down experiment, recombinant WT and R164Q Ykt6 SNARE motifs bound to the Syx17 SNARE domain with similar affinity **(Fig 5G)**. To test the interaction between HOPS and the zero layer mutant Ykt6, we performed another GST pulldown by using GST tagged Ykt6[WT] and Ykt6[R164Q] full length proteins and a lysate from Myc-Dor/Vps18 expressing larvae. Equal amounts of the HOPS core subunit Myc-Dor/Vps18 were seen in WT and R164Q Ykt6-GST pulldowns. Most importantly, endogenous Car/Vps33A, the HOPS SNARE motif binding subunit, was also obviously detected in both WT and R164Q Ykt6-GST pulldowns **(S7C Fig)**.

These data suggest that while membrane association is required, the zero layer arginine is dispensable for Drosophila Ykt6 function and further confirms our hypothesis that Ykt6 acts as a non-conventional SNARE in the course of autophagosome-lysosome fusion. These findings fit well with the study of Meiringer and colleagues, in which they showed that yeast Sec18/Nsf more efficiently removes Ykt6 than Sec22 from a SNARE complex consisting of the same Qabc SNAREs [47].

## Discussion

In this work, we demonstrated that the R SNARE Ykt6 is essential for autophagosome-lysosome fusion and it is present on lysosomes and autophagic vesicles in Drosophila. We also showed that Ykt6 can form a SNARE complex with Syx17 and Snap29, but this complex is less stable than the Syx17-Snap29-Vamp7 bundle. These findings, together with our observation that the zero layer mutant Ykt6 is fully functional unlike in yeast, raise the possibility that Ykt6 forms an only partially zippered SNARE complex, perhaps due to incompatibility between the C-terminal parts of Ykt6 and Syx17 SNARE motifs. Recently, stabilization of the C-terminal region of the assembled SNARE bundle was shown to be required for vesicle fusion [54]. Based on the differences between Vamp7 and Ykt6 in structure prediction and their relative binding affinity to Syx17 and Snap29, it seems unlikely that Syx17-Snap29-Ykt6 represents a classical, fusion-competent SNARE complex.

The N-terminal part of Syx17 as well as the longin domains of Ykt6 and Vamp7 all bind to the HOPS central subunit Dor/Vps18 but not to Car/Vps33A (which may only bind SNARE domains), suggesting that these HOPS-SNARE interactions may stabilize and proofread the forming SNARE complexes, and that SNAREs present on different vesicles may promote tethering by recruiting HOPS. The cycling of Ykt6 between vesicles and the cytosol makes Ykt6 an excellent candidate as a key regulator of vesicle tethering and fusion. The requirement of two R SNARES for the same fusion event could also be explained by the possible redundancy between Vamp7 or Ykt6, but a full block of autophagosome clearance is seen in both Vamp7 and Ykt6 loss of function cells [8], and Vamp7 binds to Syx17 and Snap29 much stronger than Ykt6 does. In fact, an earlier study reported that Ykt6 dissociates from the vacuole fusion site before the actual fusion is completed [18], also suggesting that yeast Ykt6 is not functionally redundant with the other R SNARE, Nyv1. Membrane fusion requires the presence of numerous necessary factors (SNAREs, tethers, small GTPases, etc.) at the same time. As Ykt6 is highly mobile and can shuttle between vesicles and the cytosol, it may temporarily stabilize the Syx17-Snap29 Qabc SNARE complex bound to HOPS until the appropriate transmembrane R SNARE Vamp7 enters the fusion site. In line with this, we demonstrated that overexpression of Vamp7 restores autophagosome-lysosome fusion in Ykt6 loss-of-function cells. It is also worth noting that a cytosolic, soluble pool of human Syntaxin 17 has been identified thanks to glycine zipper formation between the two transmembrane domains [10], so even a fully formed, cytosolic Syx17-Snap29-Ykt6 SNARE complex might be identified in future biochemical and cell fractionation studies.

Other multisubunit tethering complexes such as GARP, Dsl1 or COG also bind to various SNARE complexes, and the Dsl1 complex interacts with the NTDs of Sed5 and Sec20 SNARE proteins [47,55–57]. Thus, SNARE complexes may have two distinct roles during membrane fusion: to facilitate docking by taking part in the recruitment of multisubunit tethers, and to mediate fusion by SNARE zippering.

**Fig 6:**
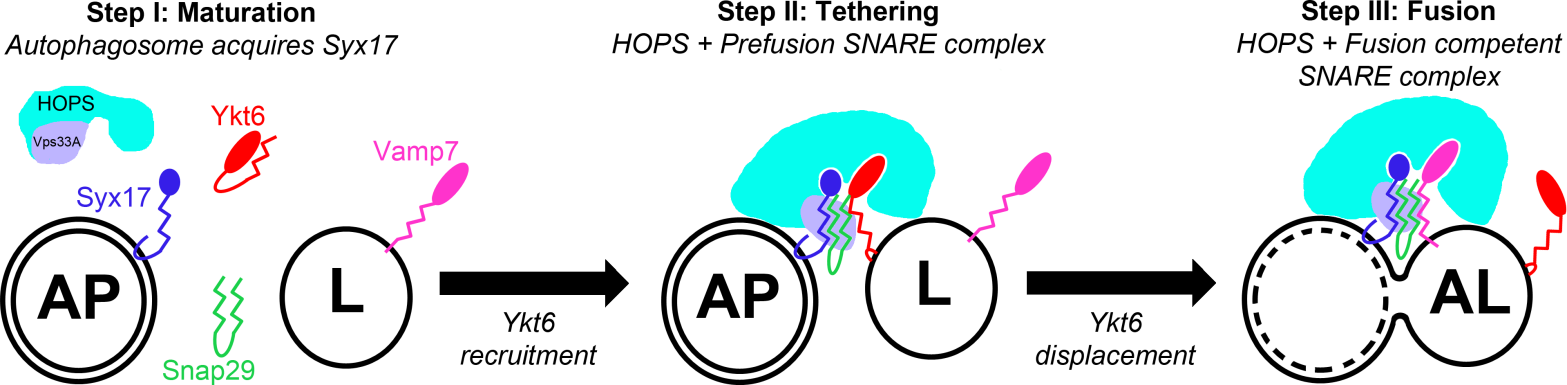
A possible model of Ykt6 function during autophagosome-lysosome fusion. In our proposed model we divide the process of autophagosome-lysosome fusion into three main steps. During the “Maturation” step, nascent autophagosomes gain fusion competence by acquiring Syx17. At the “Tethering” stage, soluble Ykt6 is recruited and associates with the lysosomal membrane. Concomitantly, Ykt6 may form a not fully zippered, fusion incompetent SNARE complex with Syx17 and Snap29, which may provide multiple surfaces for HOPS interaction and recruitment. We hypothesize that in the final “Fusion” stage, Vamp7 displaces Ykt6 and forms a stable, fusion competent SNARE complex with Syx17 and Snap29 to achieve membrane fusion.

Based on our findings, we propose a possible scenario for autophagosome-lysosome fusion **(Fig 6)**: first, Syx17 (and together with it Snap29) is recruited to autophagosomal membranes. Second, a not fully zippered, “pre-fusion” SNARE complex is assembled with Ykt6, which is possibly bound to lysosomes. Third, HOPS is recruited with the help of multiple SNARE interactions, involving both the N-terminal regions and SNARE domains, to promote tethering. Finally, the appropriate R SNARE Vamp7 arrives to the fusion site, displaces Ykt6, and forms the fusion competent SNARE complex together with Syx17 and Snap29.

It is also worth pointing out that Ykt6 is involved in multiple types of intracellular vesicle fusion events, so the fusion regulatory activity of Ykt6 is likely broader and not restricted to autophagosome-lysosome fusion. The facts that a zero ionic layer mutant form of Ykt6 is fully functional during autophagy and that R164Q mutant animals are completely viable suggest that forming a fusion-competent SNARE complex may not be the main function of Ykt6 during vesicle tethering and fusion in Drosophila.

## Materials and methods

### Fly work

Flies were raised on standard corn meal-agar-yeast medium. For starvation experiments, early L3 stage larvae were kept in 1.5 ml tubes containing 20% sucrose solution at 25°C for 4 h. Experimental genotypes are listed in the **S1 Table**.

*ykt6[G0155]*; *ykt6[A]*; *ykt6[C]*; *actin-Gal4 (Act5C-Gal4)*; *Hsp70-Gal4*, *Syx5[AR113]*, *Vamp7[G7738]* (*Vamp7[EP]* in the text) and *ykt6*^*RNAi HMJ21032*^ (*ykt6 RNAi/2* in the text) Drosophila lines were obtained from the Bloomington Drosophila Stock Center (Bloomington, IN). The *ykt6*^*1515R-1*^ (*ykt6 RNA/*1 in the text), *Syx5*^*4214R-4*^, and *Vamp7*^*1599R-1*^ RNAi lines were obtained from NIG-Fly (National Institute of Genetics, Mishima, Japan). *3xmCherry-Atg8a*, *Lamp-3xmCherry*, tubulin promoter-driven *GFP-p62* and *UAS-myc-dor* fly lines were described [4,58]. *UAS-Lamp1-GFP* [59] line was a kind gift of Helmut Kramer. UAS and r4 promoter regulated *mCherry-Atg8a* [60], *FB-Gal4* and *hs-Flp*, *FRT19A ubi-RFP/GFP* flies were gifts from Thomas Neufeld. UAS-GFP-Vamp7 flies [61] were obtained from Amy Kiger. We used genomic promoter driven *ykt6[WT]*; *ykt6[R164Q]*, *ykt6[C195A]*, *ykt6[C196A]* transgenic flies for rescue experiments and *UAS-3xHA-ykt6* for immunostainings (all generated in this study).

For clonal analysis of *ykt6* RNAi, Gal4 expressing GFP positive fat cell clones were generated randomly using hs-Flp; Act>CD2>Gal4, and induction of GFP negative, homozygous mutant mitotic clones was performed as described [62]. Syx5 mutant fat cell clones were generated by a previously described positive marking clonal strategy [4].

### Fluorescent imaging and immunostainings

For LTR staining, fat tissue of early L3 larvae was dissected and incubated in a drop of PBS containing 0.2 μM LysoTracker Red (Invitrogen) for 5 min at 25°C. Then the samples were washed once in PBS and mounted in 0.2 μg/ml DAPI and 50% glycerol in PBS. For transient overexpression of HA-Ykt6 in *Vamp7* mutant cells, *UAS-3xHA-ykt6; Vamp7[EP]/Vamp7[EP]; Hsp70-Gal4/+* early L3 stage larvae were heat shocked for 1h in a 37°C water bath and processed for LTR staining after 6h recovery.

For immunostainings, early L3 larvae were bisected in PBS and fixed for 50 min in 4% paraformaldehyde in PBS at 25°C. Samples were rinsed 4x and washed 2x for 15 min in PBS. After a 20 min wash in PBS containing 0.1% TritonX-100 and 0.05% sodium deoxycholate (PBTX-Doc), the samples were blocked in 5% FCS in PBTX for 30 min. Then the samples were incubated in first antibody solution (primary antibodies dissolved in blocking solution) overnight at 4°C. Samples were washed once in 4% NaCl PBTX-Doc for 15 min, rinsed 3x in PBTX-Doc, and washed 2x in PBTX-Doc for 15 min at 25°C. Samples were blocked again with 5% FCS PBTX-Doc and incubated in secondary antibody solution for 4 h at 25°C. Then specimens were washed in PBTX-Doc containing DAPI and 4% NaCl for 15 min, rinsed 3x and washed 2x for 15 min in PBTX-Doc. Detergent was washed out by rinsing 3x and washing 2x for 15 min with PBS. Finally, fat tissues were dissected and mounted in Vectashield (Vector Labs). The following primary antibodies were used: monoclonal rat anti-HA (1:80; Roche, 3F10), polyclonal rabbit anti-Cathepsin L (1:100; Abcam, ab58991), polyclonal rabbit anti-dsRed (1:500, Clontech, 632496). Secondary antibodies: Alexa488-conjugated anti-rat (1:1000, Life Technologies), Alexa568-conjugated anti-rabbit (1:1000, Life Technologies).

Images were obtained with an AxioImager.M2 fluorescent microscope equipped with an ApoTome2 confocal unit using EC Plan-Neofluar 40×/0.75 Air or Plan-Apochromat 40×/0.95 Air objectives and AxioVision 4.82 or ZEN 2.3 softwares (all Zeiss). Images were processed in ZEN Lite (Zeiss) and Photoshop (Adobe).

### Electron microscopy and immunogold labeling

Genetically mosaic L3 larvae containing GFP negative *ykt6[G0155]* or GFP positive *Syx5[AR113]* mutant clones were dissected in a drop of PBS, and the fat tissues were stuck on the surface of a poly-L-lysine coated coverslip. After fluorescent imaging of the clone cells, the samples were fixed in 1% glutaraldehyde, 1% sucrose, and 0.028% CaCl_2_ in 0.1 N sodium cacodylate overnight at 4°C, and postfixed in 0.5% osmium tetroxide for 1 h at 25°C. Then samples were embedded in Durcupan (Fluka) by following the manufacturer’s instructions. The area containing the mutant cells was identified in the blocks containing embedded fat tissue in a stereomicroscope based on the fluorescent image. Semi-thin sections were cut and stained by toluidine blue to identify mutant cells, as described [4]. Ultrathin sections were then cut, stained with Reynold’s lead citrate and used for obtaining images with a JEM-1011 transmission electron microscope (JEOL) equipped with Morada camera and iTEM software (both Olympus).

For immuno-EM analysis of HA-Ykt6, we used a rabbit polyclonal anti-HA primary (1:10, Sigma) and 10 nm gold-conjugated goat anti-Rabbit (1:100; Sigma) secondary antibodies. The labeling procedure was performed as described [63].

### Statistics

Always the original unprocessed images were used for quantification. Fluorescent dots were quantified by using ImageJ (NIH). The same person set every threshold in all images of a given type of experiment. In clonal experiments a randomly generated RNAi or mutant cell clone and a neighboring control cell pair was chosen for quantification. As ykt6 loss of function cells are usually smaller than the controls, the data pairs were corrected by the cell size ratio. For area based quantification of LTR staining, dots were counted from a randomly chosen 300 x 300 pixel area from original images. For colocalization analyses 200 dots were counted randomly from 10 images per experiment. The Gaussian or non-Gaussian distribution of the datasets was checked by SPSS (IBM) software. For statistical analyses, t-tests were performed when two Gaussian datasets were compared and u-tests if at least one of the two datasets was non-Gaussian. Multiple comparisons were carried out for Fig 5F (ANOVA) and S6D Fig (Kruskal-Wallis test). Error bars show standard error. All numerical data underlying graphs or summary statistics are shown in **S3 Table**.

### Molecular cloning and generation of transgenic Drosophila

For cloning UAS-3xHA-ykt6, the coding region of *ykt6* was PCR amplified from Drosophila cDNA template and cloned into a pUAST-3xHA vector as a NotI-Acc65I fragment. UAS-Syx17-3xFLAG (lacking TM domains) UAS-3xHA-Snap29, UAS-3xHA-Vamp7 (lacking TM domain) were described earlier [8].

For pulldowns, SNARE proteins/fragments were cloned into pETARA or/and pETMBP vectors [64], which contain C-terminal Glutation S-tranferase/Maltose Binding Protein tag and C-terminal hexahistidine-tag, respectively, using BamHI and XhoI restriction sites. Ykt6 full length, ykt6 longin domain and VAMP7 longin domain were cloned into pET21c vector (which contains C-terminal Glutation S-transferase and hexahistidine tags) using InFusion HD Cloning Kit (Clontech) [64]. Zero-layer mutant R164Q Ykt6 constructs were generated using QuickChange (Agilent) site-directed mutagenesis.

For expressing His-tagged Drosophila Ykt6 protein, which was used for generating the anti-Ykt6 antibody, the entire coding region of *Ykt6* was amplified from cDNA and inserted into pBH4 vector using BamHI-SalI cloning.

For generating endogenous promoter driven Ykt6[WT] rescue transgenes, ykt6 genomic locus was PCR amplified and cloned into pCasper5 vector using NotI-BglII. Ykt6 R164Q, C195A and C196A mutations were generated by using overlapping mutant oligo pairs. All primers used in this study are listed in **S2 Table**. Transgenic flies were generated by microinjection of pCasper5-ykt6[WT], [R164Q], [C195A], [C196A] and pUAST-3xHA-ykt6 plasmids into embryos (BestGene).

### Biochemistry and antibody preparation

Co-IPs were carried out by transient expression of SNARE proteins in D.Mel-2 Drosophila cells. Cell lysates were loaded onto anti-FLAG affinity gel beads (Sigma-Aldrich, A2220), and the interactions were detected by western blot as described earlier [8].

For pulldown experiments, recombinant SNARE constructs were expressed overnight at 18°C in E. coli Rosetta(DE3) pLysS (Novagen) cells induced with 0.1 mM IPTG at OD 0.6–0.7. Cells were then centrifuged and suspended in lysis buffer (pH 8.0, 50 mM Na_2_HPO_4_, 300 mM NaCl, 20mM imidazole, 0.1% Triton-X, 5 mM-β-mercaptoethanol, protease inhibitors). Lysed samples were centrifuged at 48.000g for 30 minutes. Ni-NTA resin was added in the supernatant and incubated for 30 minutes at 4°C. Beads were washed with washing buffer (pH 8.0, 50 mM Na_2_HPO_4_, 1 M NaCl, 40mM imidazole, 0,1% Triton-X, 5 mM β-mercaptoethanol) and were eluated in Elution buffer (pH 8.0, 20 mM Tris, 200 mM NaCl, 400 mM imidazole, 10% glicerol, 0,1% Triton-X, 5 mM-β-mercaptoethanol) used for pulldown assays. Prey proteins for pulldown experiments were purified with further MBP affinity chromatography using standard protocols. All resins were from GE Healthcare.

For GST pulldown assays, the glutation resin (New England BioLabs) was first equilibrated with binding buffer (20 mM Tris, 50 mM NaCl, 0.1% Triton-X, 2 mM β–mercaptoethanol), then 0.5 mg GST fused SNARE proteins (and GST as negative control) were immobilized on it. In the binding experiments, 40 μl of resin saturated with baits were incubated in the presence of 20 mg fly lysates or 20 μM preys in binding buffer (200 μl total volume) for 30 min at 4°C. Glutation beads were pelleted with centrifugation (2000 g for 2 min) and washed 3x with 20 mM Tris, 300 mM NaCl, 0.1% Triton-X, 2 mM β–mercaptoethanol. Retained proteins were eluted from the resin with SDS loading buffer. Samples were subjected to SDS-PAGE and stained with Coomassie protein dye to validate load ratio, and interactions were detected by western blot.

For anti-Ykt6 antibody production, His-Ykt6 expression constructs were transformed into BL21(DE3)Rosetta cells. Protein production was induced with 0.5 mM IPTG (3h, 37°C, 250 rpm shaking), cells were lysed in 6 M Guanidine HCl lysis buffer and the cleared lysate was loaded onto Ni Sepharose excel column (GE Healthcare). His6-tagged proteins were purified under denaturing condition according to the manufacturer’s instructions, the His_6_-Ykt6 protein was used to immunize female Wistar rats. Immunoglobulin fraction of crude rat sera was obtained by ammonium-sulfate precipitation, and the polyclonal anti-Ykt6 antibody was further purified on nitrocellulose bound recombinant protein as described [65].

To prepare whole larval western blot samples, 4 h starved early L3 larvae were dried with filter paper, collected in plastic tubes and weighed. After addition of 1:1 PBS:Laemmli buffer (20 μl/mg larvae), samples were boiled for 5 min and homogenized. Then samples were boiled for 5 min again, and centrifuged with 13300 rpm for 5 min. The supernatants were collected and stored at -20°C. Western blots were carried out by following standard protocols and as described earlier [8]. The following primary antibodies were used: monoclonal rat anti-HA (1:2000 Roche, 3F10), monoclonal mouse anti-FLAG (1:2000, Sigma-Aldrich, M2), monoclonal mouse anti-Myc (1:2000, Sigma-Aldrich, M4439), polyclonal rabbit anti-Vamp7 (1:300, gift from Andrew Peden [26]), polyclonal rat anti-Ykt6 (1:300, this study). Polyclonal rat anti-Syx17 and rat anti-Snap29, rabbit anti-Atg8a and rabbit anti-p62 (all 1:5000) were described in our earlier studies [8,66]. Polyclonal antibodies for HOPS subunits were rabbit anti-Car (1:1000)[67], rabbit anti-Vps16A (1:2000)[59] (gifts from Helmut Kramer). Secondary antibodies: alkaline phosphatase conjugated anti-rabbit, anti-rat and anti-mouse (all 1:5000, EMD Millipore). Blots were developed with NBT/BCIP chromogenic substrate. For western blots aiming to measure Ykt6 and Vamp7 protein levels, IRDye 800CW conjugated goat anti-rat and IRDye 800CW goat anti-rabbit (LI-COR) fluorescent secondary antibodies (both 1:15,000) were used. Signals on these blots were detected using an Odyssey CLx near-infrared laser scanning fluorescence imaging system (LI-COR).

### 3D homology modeling

3D homology model of putative fruit fly autophagic SNARE complexes were built by SWISS-Model software [68], using the only available high resolution crystal structure (1.4 Å) of the directly relevant human SNARE complex (PDB code: 4WY4) as template. Each SNARE motif (VAMP7: 125–185 aa; ykt6: 139–199 aa; SNAP29 SN1: 75–137 aa; SNAP29 SN2: 219–281 aa; Stx17: 156–218 aa) sequence was downloaded from NCBI and used individually for sequence alignment. All SNARE motif models were built based on their human homologs, respectively. Structure energy minimization was done by Amber function in CHIMERA 1.11.2 software.

## Supporting information

S1 Fig Loss of Ykt6 leads to accumulation of autophagosomes. (A, B) RNAi knockdown of *ykt6* using two independent RNAi transgene in fat cell clones (GFP+) leads to accumulation of small 3xmCherry-Atg8a dots, unlike the big bright structures seen in the surrounding control cells (GFP-). Please see Fig 4H and S6F Fig for quantification of data in panels A and B, respectively. (C-F) Fat cell clones (GFP-) homozygous for the *ykt6[A]* (C) or *ykt6[C]* (E) mutant alleles also accumulate small mCherry-Atg8a autophagic structures compared to GFP+ controls cells. *ykt6* loss of function cells are encircled in grayscale panels in A-C, E, G. (D, F) Quantification of data from C (D) and E (F); n = 10. (G) A striking accumulation of the selective autophagy cargo p62 is obvious in *ykt6[G0155]* mutant cells (marked by lack of RFP), compared to surrounding control fat cells from a well-fed larva. Quantification of data from G (H), n = 10. Scale bar in A is 20 μm for A-C, E, G.
(TIF)

S2 Fig Ykt6 is indispensable for autophagosome-lysosome fusion. (A) Knockdown of *ykt6* prevents the colocalization of the autophagy reporter 3xmCherry-Atg8a with the lysosomal marker Lamp1-GFP. Inset shows the boxed region enlarged. (B) The autophagic marker 3xmCherry-Atg8a shows extensive colocalization (yellow arrowheads) with the lysosomal protease Cathepsin-L in GFP+ control cells. In contrast, there is no overlap between these two markers in *ykt6[G0155]* mutant cells (GFP-), indicating that these cells are defective in autophagosome-lysosome fusion. Bottom panels show the boxed region enlarged, and *ykt6* loss of function cells are encircled in grayscale panels. Scale bars: 20 μm.
(TIF)

S3 Fig Loss of Syntaxin 5 does not prevent autophagosome-lysosome fusion. (A) Knockdown of Syx5 in GFP-marked cells decreases fat cell size but does not prevent the formation of big, bright 3xmCherry-Atg8a positive autolysosomes. Right panel: quantification of data, n = 10. Syx5 loss-of-function cells are encircled in grayscale panels in A and B. (B) Autolysosomal LTR staining is strongly enhanced in GFP-positive Syx5 mutant clones compared to neighboring control fat cells. (C) Ultrastructural analysis of Syx5 mutant fat cells clearly identifies the presence of autolysosomes (AL) containing partially degraded cargo. Scale bars: 20 μm in A, B, 1 μm in C.
(TIF)

S4 Fig Additional Ykt6 and Vamp7 expression data. (A) HA-Ykt6 shows a mostly diffuse cytosolic pattern in fat cells of well-fed animals, showing no obvious overlap with endogenous Cathepsin L punctae. (B) Ykt6 partially overlaps with lysosomal Cathepsin L dots in Vamp7 mutant fat cells. Inset shows the boxed region enlarged. (C) Quantification of Cathepsin L data from panels A, B and Fig 2A. (D, E) Western blot data. The protein level of Ykt6 is decreased in *ykt6* knockdown larvae, validating both our new antibody and the RNAi efficiency (D). Starvation induces no change in the level of Ykt6 and Vamp7 proteins, respectively (E). Scale bar: 20 μm for A, B.
(TIF)

S5 Fig Additional SNARE interaction data. (A, B) Biochemical pulldown data from Drosophila lysates. Full-length, recombinant Ykt6 pulls down endogenous Syx17 from Drosophila lysate (A), but it shows no binding to overexpressed GFP-Vamp7 (B). (C) Predicted 3D model of putative Drosophila autophagic SNARE complexes, with overlaid Vamp7 and Ykt6 indicated in green and pink, respectively. (D-G) Residues with different charge and shape in the corresponding positions of Vamp7 and Ykt6 suggest weaker Syx17-Ykt6 interaction compared to Vamp7-Syx17. Based on the predictions it is likely that arginine 172 in Vamp7 can form two H-bonds (dashed lines) with asparagine 207 and glutamine 210 of Syx17 (D), while the corresponding tyrosine 186 in Ykt6 cannot (E). Alanine 179 in Vamp7 is small enough to fit with the opposing asparagine 214 residue in Syx17, which is compatible with the formation of a tight SNARE bundle (F). In contrast, Ykt6 carries a larger asparagine in the corresponding 193th position (red arrow), which collides with the backbone of asparagine 214 in Syx17 (G), likely causing extra tension in the SNARE bundle. Please note that protein surfaces are indicated only for Vamp7 and Ykt6 in F and G, respectively.
(TIF)

S6 Fig Additional genetic interaction data between Ykt6 and Vamp7. (A-C) Autolysosomal LTR staining of fat tissues from starved larvae. Punctate starvation-induced LTR staining seen in control animals (A) is blocked in *Vamp7* mutants (B). Overexpression of Ykt6 fails to restore LTR dot formation in *Vamp7* mutants (C). Quantification of LTR data from A-C (D), n = 10. Co-overexpression of GFP-Vamp7 restores the size of autophagic 3xmCherry-Atg8a puncta in GFP-marked cells expressing an independent *ykt6* RNAi line (E, compare to S1B Fig), too, similar to the experiments shown in Fig 4E and 4G. Quantification of data from E and S1B Fig (F), n = 10. Overexpression of GFP-Vamp7 restores 3xmCherry-Atg8a puncta size in *ykt6[G0155]* mutant fat cells (G). The mutant cells are identified by the lack of nuclear GFP signal in the rightmost panel showing increased brightness of the GFP channel, where asterisks label the nuclei of control cells. Note that GFP-Vamp7 colocalizes with autophagic 3xmCherry-Atg8a in both mutant and control cells in panels E, G. Scale bar: 20 μm for A-C, E, G.
(TIF)

S7 Fig Analysis of biochemical interactions between SNAREs and HOPS. (A) GST-tagged recombinant Ykt6 full length (Ykt6FL), Ykt6 longin domain (Ykt6LD) and the N-terminal domain of Syx17 (Syx17NTD) all bind to overexpressed myc-Dor/Vps18 (a core HOPS subunit) in GST pulldown experiments from larval lysates. In contrast, only the SNARE motif-containing Ykt6FL protein binds to endogenous Car/Vps33A, the SNARE chaperoning subunit of HOPS. GST serves as a negative control. (B) Recombinant full length Ykt6 (Ykt6FL), and the longin domains of Ykt6 or Vamp7 (Ykt6LD or Vamp7LD, respectively) were immobilized on glutathione resin and the lysate of Myc-Dor/Vps18 expressing L3 larvae was added. The HOPS core subunit Myc-Dor/Vps18 bound to both Ykt6LD and Vamp7LD, as well as Ykt6FL. White asterisk marks a degradation product in the Coomassie-stained gel. (C) Both the wild type and R164Q mutant forms of Ykt6-GST pull down Myc-Dor/Vps18 and endogenous Car/Vps33A in similar amounts from larval lysates.
(TIF)

S1 Table List of the detailed genotypes used for experiments. (XLSX)

S2 Table List of primers used for molecular cloning. (XLSX)

S3 Table Numerical data underlying graphs or summary statistics. For each figure panel showing quantified data (Statistics panel), the Source panel(s), statistical test(s), p values, significance and raw data (including the averages, standard deviations and standard errors) are shown. Please note that puncta number values were corrected according to the cell size difference seen between control and ykt6 mutant, ykt6 RNAi/2 and Syx5 RNAi cell clones (because these cells are smaller than control cells).
(XLSX)
