# Supplementary figures and images for: Non-canonical role of the SNARE protein Ykt6 in autophagosome-lysosome fusion
Source: PLoS Genet. 2018 Apr 25;14(4):e1007359. doi: 10.1371/journal.pgen.1007359 (PMC5937789; doi:10.1371/journal.pgen.1007359)

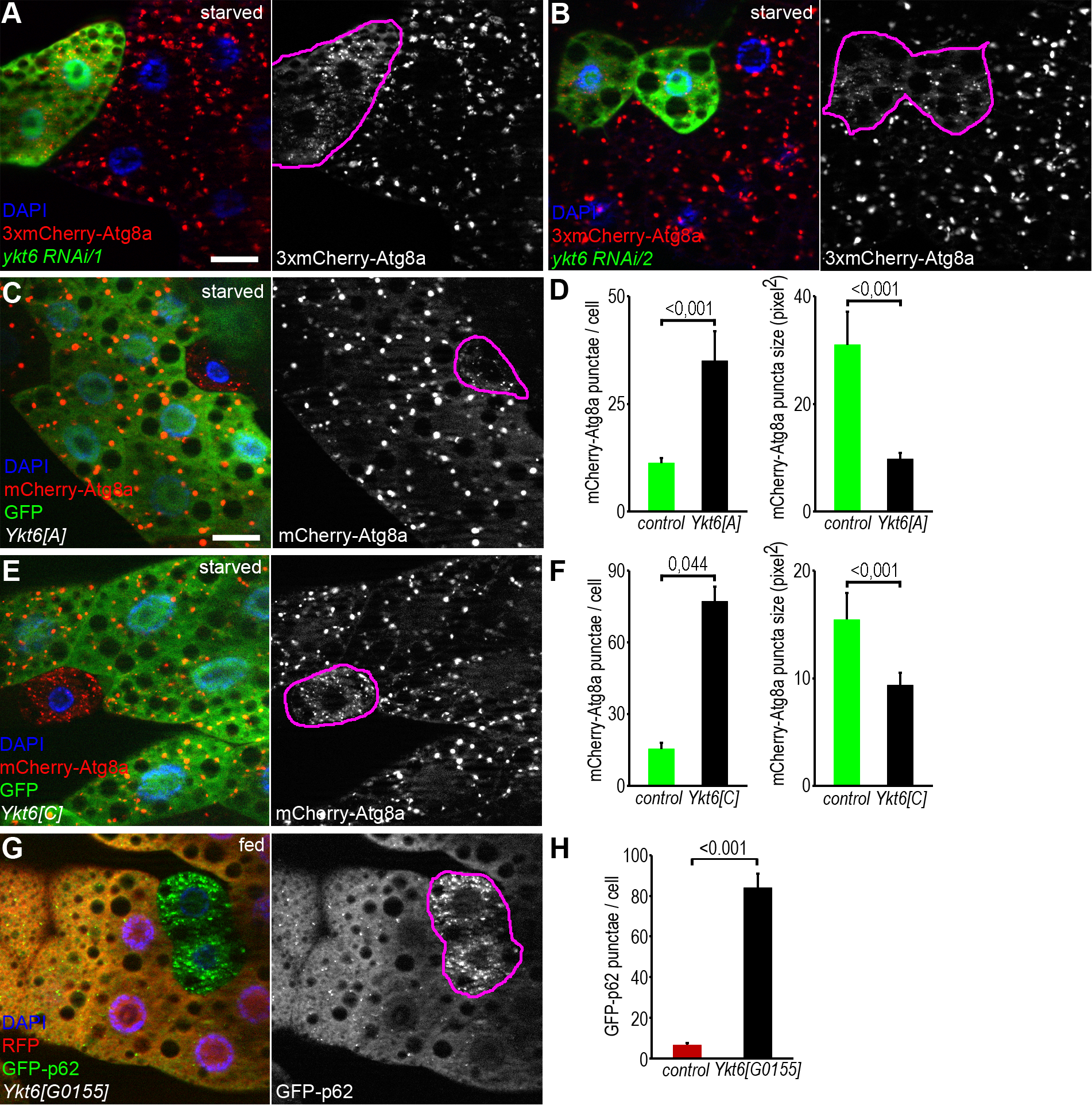

Supplement: S1 Fig — (A, B) RNAi knockdown of ykt6 using two independent RNAi transgene in fat cell clones (GFP+) leads to accumulation of small 3xmCherry-Atg8a dots, unlike the big bright structures seen in the surrounding control cells (GFP-). Please see Fig 4H and S6F Fig for quantification of data in panels A and B, respectively. (C-F) Fat cell clones (GFP-) homozygous for the ykt6[A] (C) or ykt6[C] (E) mutant alleles also accumulate small mCherry-Atg8a autophagic structures compared to GFP+ controls cells. ykt6 loss of function cells are encircled in grayscale panels in A-C, E, G. (D, F) Quantification of data from C (D) and E (F); n = 10. (G) A striking accumulation of the selective autophagy cargo p62 is obvious in ykt6[G0155] mutant cells (marked by lack of RFP), compared to surrounding control fat cells from a well-fed larva. Quantification of data from G (H), n = 10. Scale bar in A is 20 μm for A-C, E, G. (TIF) [file pgen.1007359.s001.tif]

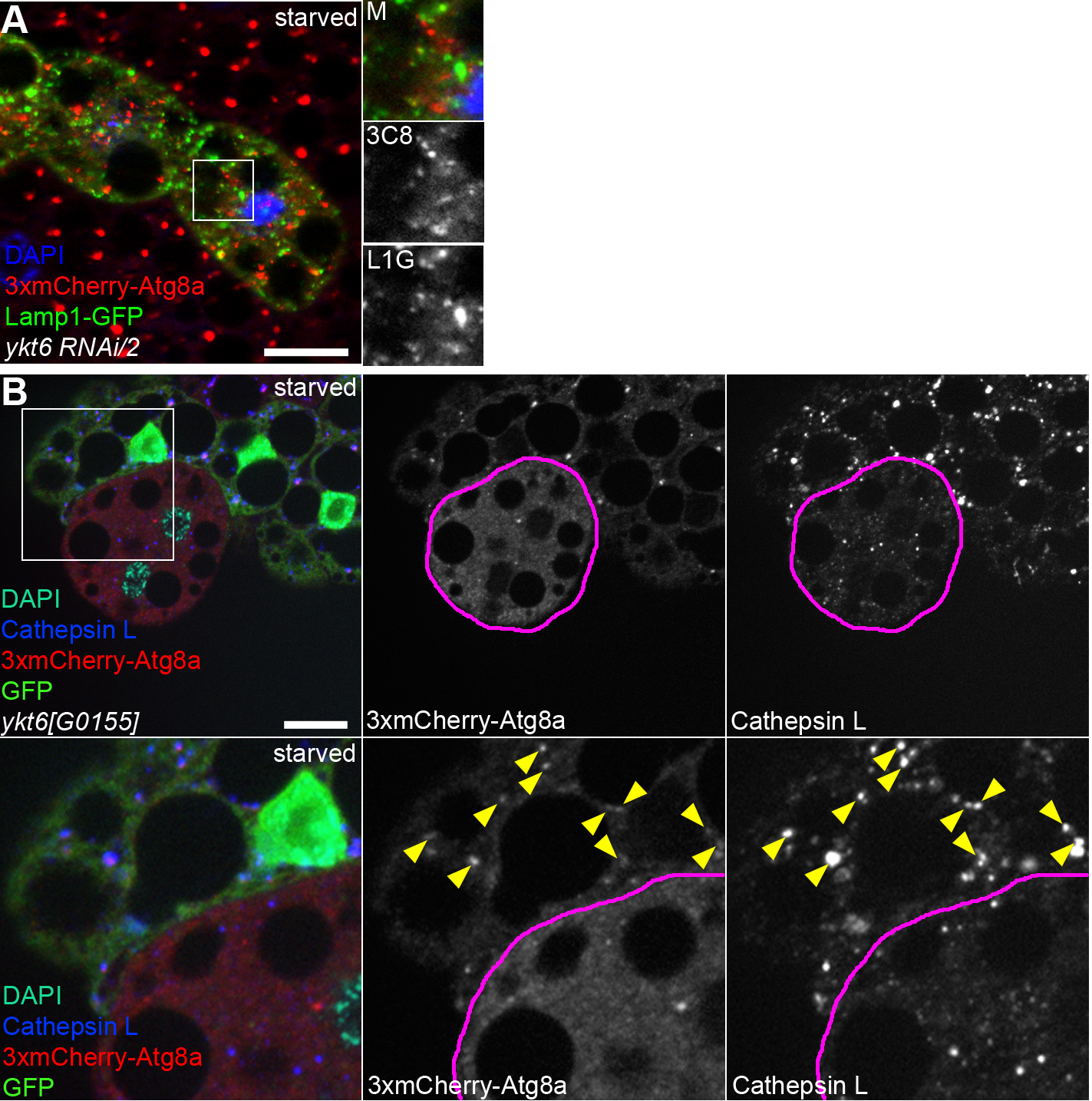

Supplement: S2 Fig — (A) Knockdown of ykt6 prevents the colocalization of the autophagy reporter 3xmCherry-Atg8a with the lysosomal marker Lamp1-GFP. Inset shows the boxed region enlarged. (B) The autophagic marker 3xmCherry-Atg8a shows extensive colocalization (yellow arrowheads) with the lysosomal protease Cathepsin-L in GFP+ control cells. In contrast, there is no overlap between these two markers in ykt6[G0155] mutant cells (GFP-), indicating that these cells are defective in autophagosome-lysosome fusion. Bottom panels show the boxed region enlarged, and ykt6 loss of function cells are encircled in grayscale panels. Scale bars: 20 μm. (TIF) [file pgen.1007359.s002.tif]

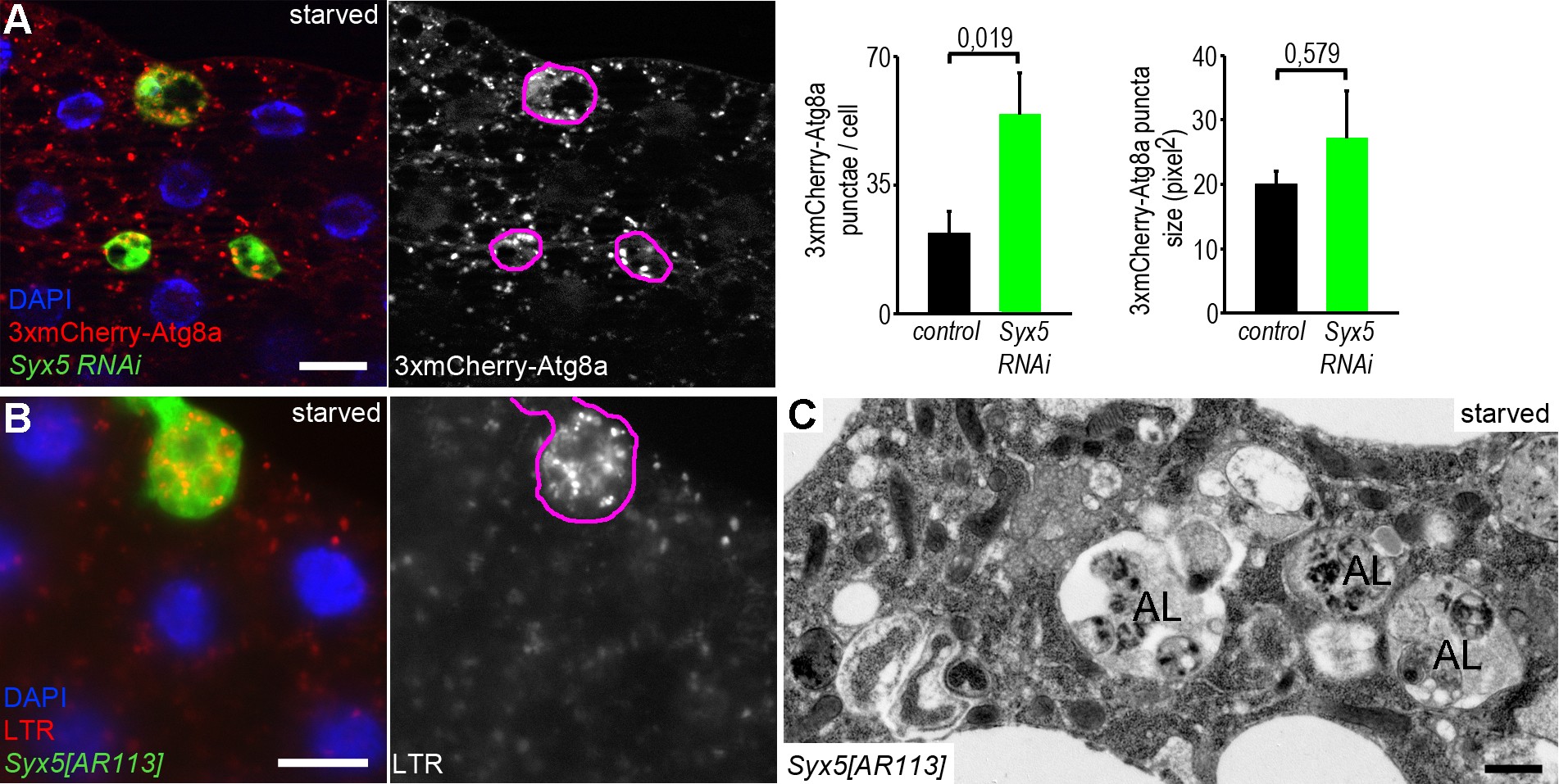

Supplement: S3 Fig — (A) Knockdown of Syx5 in GFP-marked cells decreases fat cell size but does not prevent the formation of big, bright 3xmCherry-Atg8a positive autolysosomes. Right panel: quantification of data, n = 10. Syx5 loss-of-function cells are encircled in grayscale panels in A and B. (B) Autolysosomal LTR staining is strongly enhanced in GFP-positive Syx5 mutant clones compared to neighboring control fat cells. (C) Ultrastructural analysis of Syx5 mutant fat cells clearly identifies the presence of autolysosomes (AL) containing partially degraded cargo. Scale bars: 20 μm in A, B, 1 μm in C. (TIF) [file pgen.1007359.s003.tif]

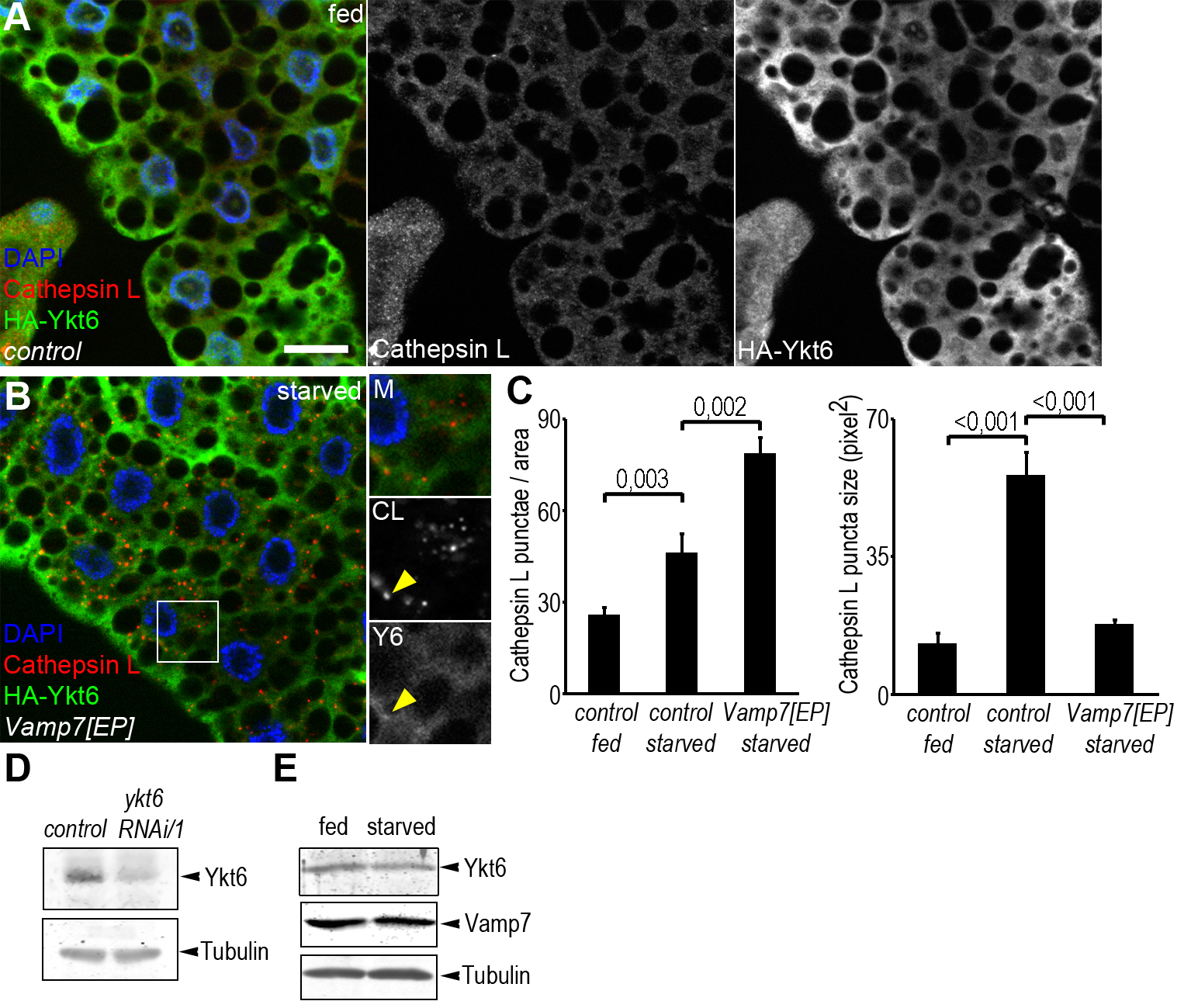

Supplement: S4 Fig — (A) HA-Ykt6 shows a mostly diffuse cytosolic pattern in fat cells of well-fed animals, showing no obvious overlap with endogenous Cathepsin L punctae. (B) Ykt6 partially overlaps with lysosomal Cathepsin L dots in Vamp7 mutant fat cells. Inset shows the boxed region enlarged. (C) Quantification of Cathepsin L data from panels A, B and Fig 2A. (D, E) Western blot data. The protein level of Ykt6 is decreased in ykt6 knockdown larvae, validating both our new antibody and the RNAi efficiency (D). Starvation induces no change in the level of Ykt6 and Vamp7 proteins, respectively (E). Scale bar: 20 μm for A, B. (TIF) [file pgen.1007359.s004.tif]

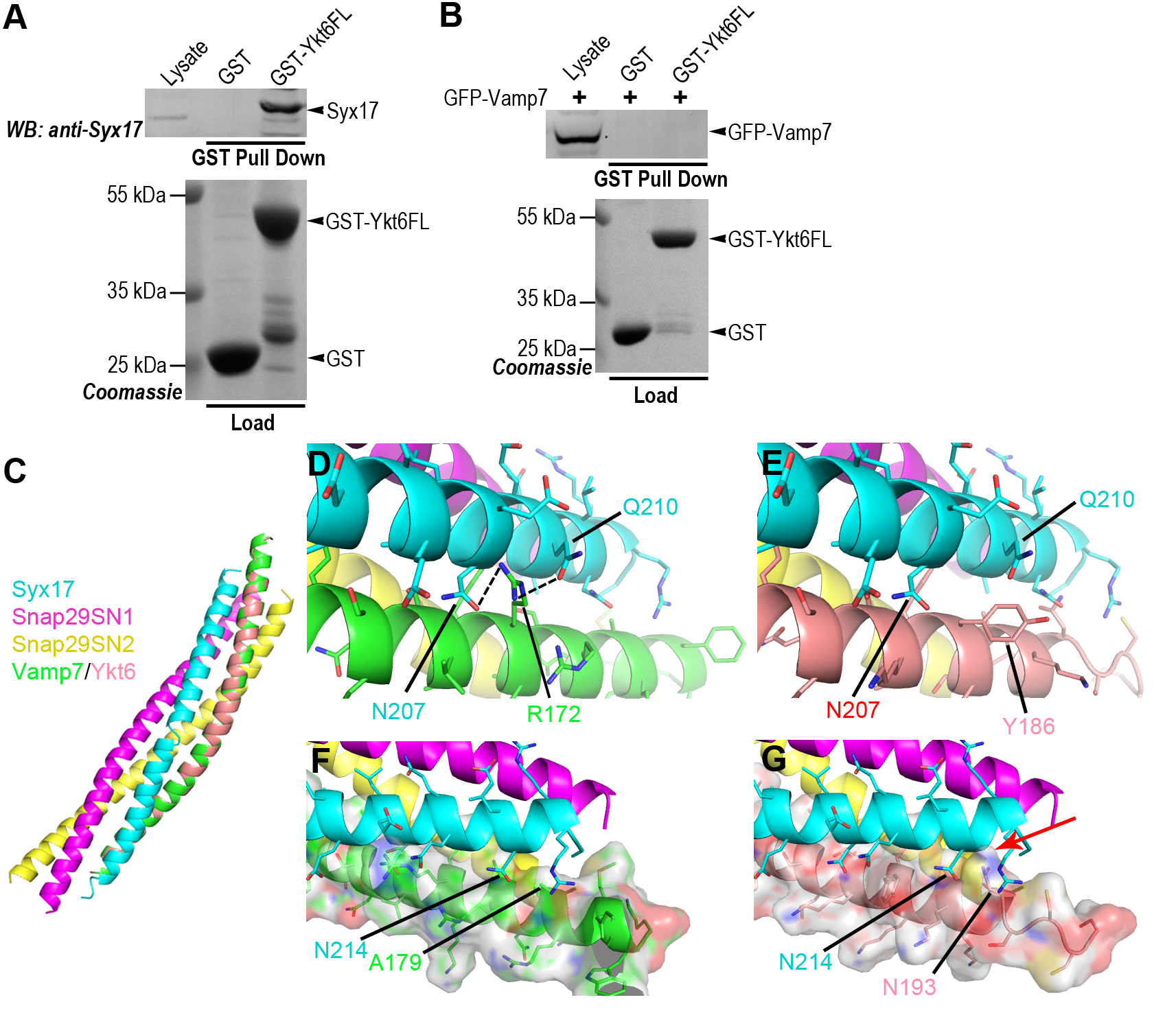

Supplement: S5 Fig — (A, B) Biochemical pulldown data from Drosophila lysates. Full-length, recombinant Ykt6 pulls down endogenous Syx17 from Drosophila lysate (A), but it shows no binding to overexpressed GFP-Vamp7 (B). (C) Predicted 3D model of putative Drosophila autophagic SNARE complexes, with overlaid Vamp7 and Ykt6 indicated in green and pink, respectively. (D-G) Residues with different charge and shape in the corresponding positions of Vamp7 and Ykt6 suggest weaker Syx17-Ykt6 interaction compared to Vamp7-Syx17. Based on the predictions it is likely that arginine 172 in Vamp7 can form two H-bonds (dashed lines) with asparagine 207 and glutamine 210 of Syx17 (D), while the corresponding tyrosine 186 in Ykt6 cannot (E). Alanine 179 in Vamp7 is small enough to fit with the opposing asparagine 214 residue in Syx17, which is compatible with the formation of a tight SNARE bundle (F). In contrast, Ykt6 carries a larger asparagine in the corresponding 193th position (red arrow), which collides with the backbone of asparagine 214 in Syx17 (G), likely causing extra tension in the SNARE bundle. Please note that protein surfaces are indicated only for Vamp7 and Ykt6 in F and G, respectively. (TIF) [file pgen.1007359.s005.tif]

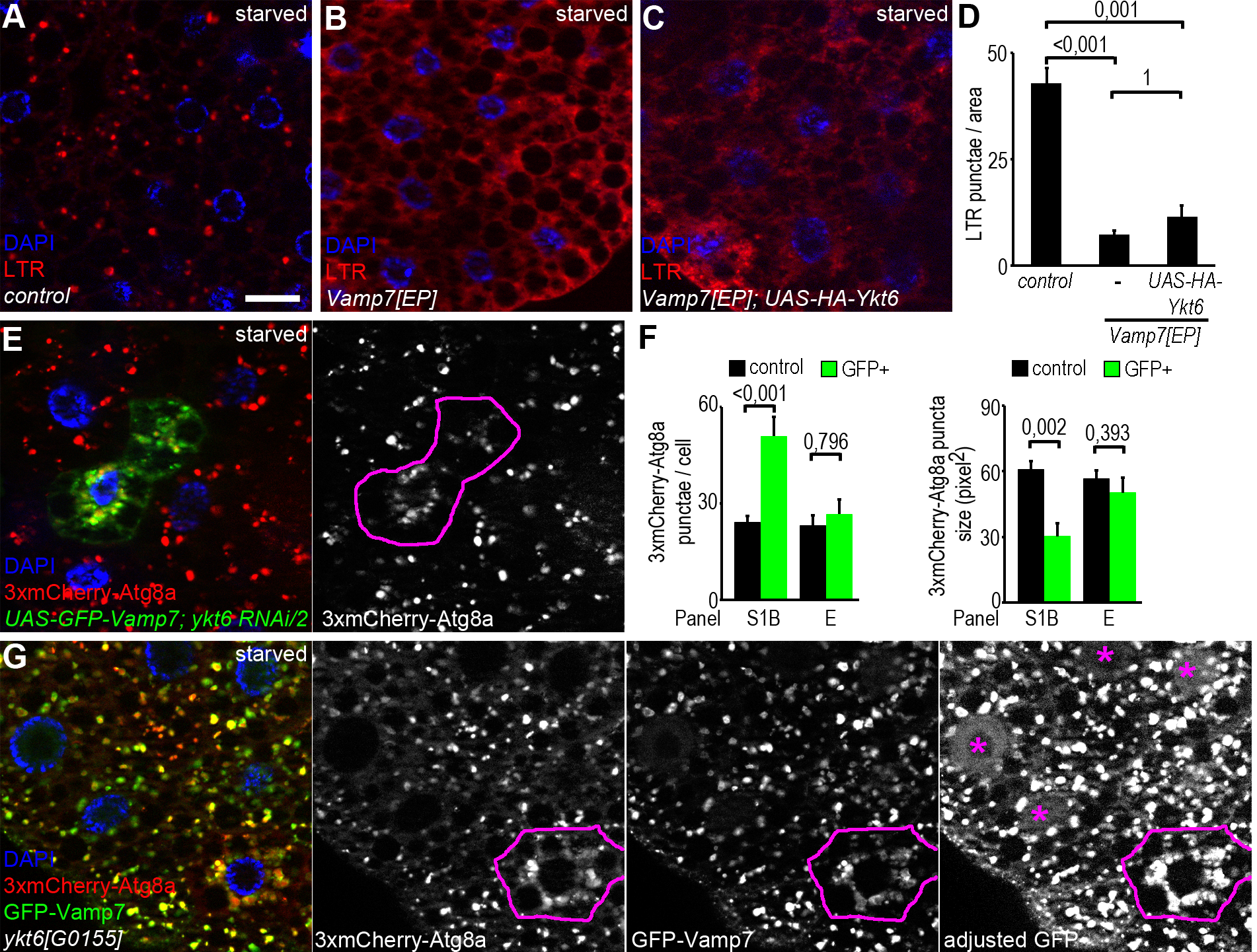

Supplement: S6 Fig — (A-C) Autolysosomal LTR staining of fat tissues from starved larvae. Punctate starvation-induced LTR staining seen in control animals (A) is blocked in Vamp7 mutants (B). Overexpression of Ykt6 fails to restore LTR dot formation in Vamp7 mutants (C). Quantification of LTR data from A-C (D), n = 10. Co-overexpression of GFP-Vamp7 restores the size of autophagic 3xmCherry-Atg8a puncta in GFP-marked cells expressing an independent ykt6 RNAi line (E, compare to S1B Fig), too, similar to the experiments shown in Fig 4E and 4G. Quantification of data from E and S1B Fig (F), n = 10. Overexpression of GFP-Vamp7 restores 3xmCherry-Atg8a puncta size in ykt6[G0155] mutant fat cells (G). The mutant cells are identified by the lack of nuclear GFP signal in the rightmost panel showing increased brightness of the GFP channel, where asterisks label the nuclei of control cells. Note that GFP-Vamp7 colocalizes with autophagic 3xmCherry-Atg8a in both mutant and control cells in panels E, G. Scale bar: 20 μm for A-C, E, G. (TIF) [file pgen.1007359.s006.tif]

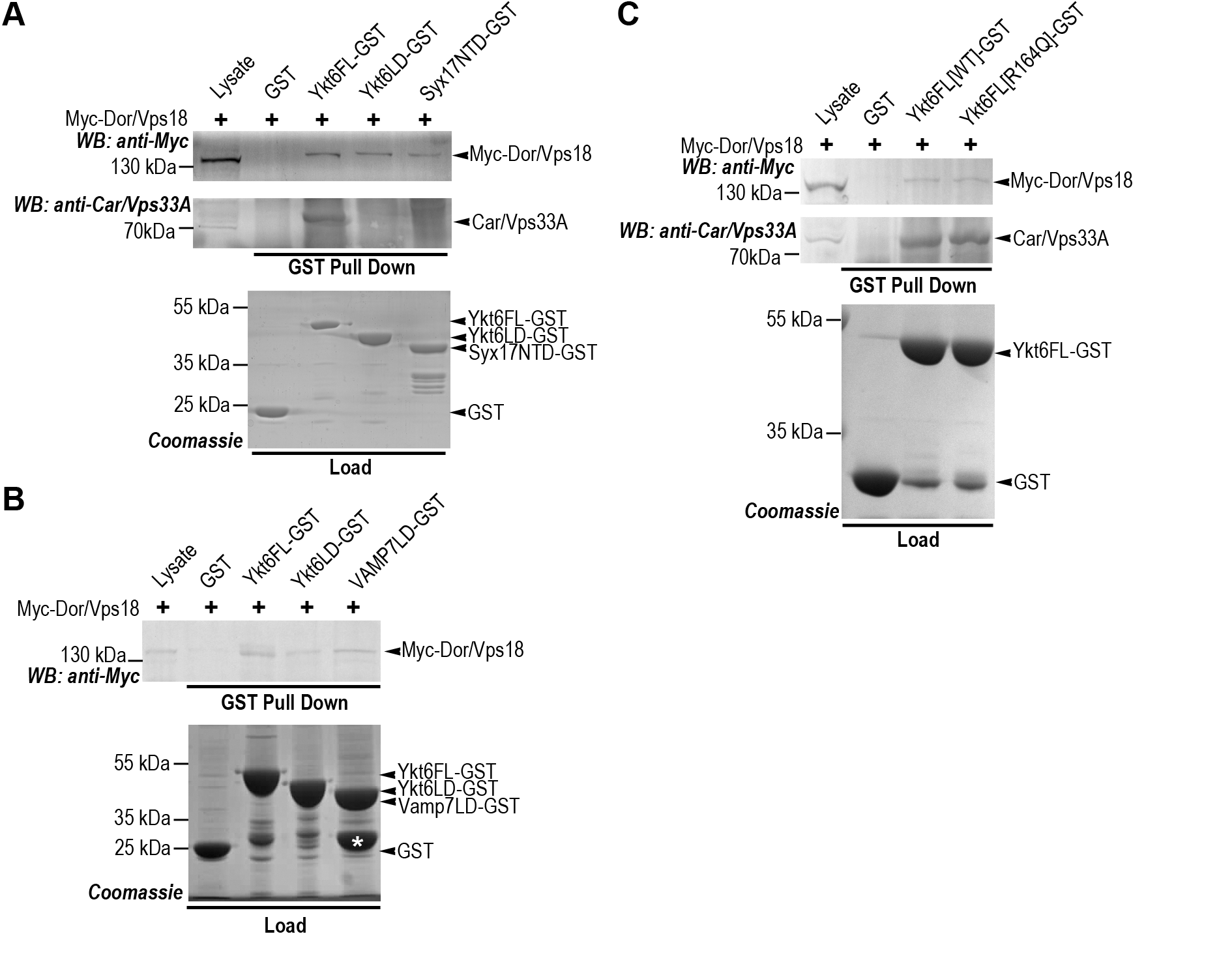

Supplement: S7 Fig — (A) GST-tagged recombinant Ykt6 full length (Ykt6FL), Ykt6 longin domain (Ykt6LD) and the N-terminal domain of Syx17 (Syx17NTD) all bind to overexpressed myc-Dor/Vps18 (a core HOPS subunit) in GST pulldown experiments from larval lysates. In contrast, only the SNARE motif-containing Ykt6FL protein binds to endogenous Car/Vps33A, the SNARE chaperoning subunit of HOPS. GST serves as a negative control. (B) Recombinant full length Ykt6 (Ykt6FL), and the longin domains of Ykt6 or Vamp7 (Ykt6LD or Vamp7LD, respectively) were immobilized on glutathione resin and the lysate of Myc-Dor/Vps18 expressing L3 larvae was added. The HOPS core subunit Myc-Dor/Vps18 bound to both Ykt6LD and Vamp7LD, as well as Ykt6FL. White asterisk marks a degradation product in the Coomassie-stained gel. (C) Both the wild type and R164Q mutant forms of Ykt6-GST pull down Myc-Dor/Vps18 and endogenous Car/Vps33A in similar amounts from larval lysates. (TIF) [file pgen.1007359.s007.tif]
